# *PETIMOT*: a novel framework for inferring protein motions from sparse data using SE(3)-equivariant graph neural networks

**DOI:** 10.1107/S2059798326006054

**Published:** 2026-07-20

**Authors:** Valentin Lombard, Julien Nguyen Van, Sergei Grudinin, Elodie Laine

**Affiliations:** a Department of Computational, Quantitative, and Synthetic Biology (CQSB), UMR 7238 IBPS, Sorbonne Université, CNRS, 75005Paris, France; b Université Grenoble Alpes, CNRS, Grenoble INP, LJK, 38000Grenoble, France; c Institut Universitaire de France (IUF) France; Institut de Biologie Structurale, France

**Keywords:** protein flexibility, motion benchmark, SE(3)-equivariant GNN, predicting linear subspaces, structural bioinformatics

## Abstract

Proteins move and deform to ensure their biological functions. Despite significant progress in protein structure prediction, approximating conformational ensembles under physiological conditions remains a fundamental open problem. This paper presents a novel perspective on the problem by directly targeting continuous compact representations of protein motions inferred from sparse experimental observations. We develop a task-specific loss function enforcing data symmetries, including scaling and permutation operations. Our method *PETIMOT* (Protein sEquence and sTructure-based Inference of MOTions) leverages transfer learning from pre-trained protein language models through an SE(3)-equivariant graph neural network. When trained and evaluated on the Protein Data Bank, *PETIMOT* shows superior performance in time and accuracy, capturing protein dynamics, particularly large/slow conformational changes, compared with state-of-the-art diffusion and flow-matching approaches, as well as traditional physics-based models. Our code and protocols are available at https://github.com/PhyloSofS-Team/PETIMOT.

## Abstract 2

We present a new formulation for protein flexibility and learn protein motions from sparse experimental data.

## 1. Introduction

Proteins orchestrate biological processes in living organisms by interacting with their environment and adapting their three-dimensional (3D) structures to engage with cellular partners, including other proteins, nucleic acids, small-molecule ligands and cofactors. In recent years, spectacular advances in high-throughput deep-learning (DL) technologies have provided access to reliable predictions of protein 3D structures at the scale of entire proteomes (Varadi *et al.*, 2024 ▸). These breakthroughs have also highlighted the complexities of protein conformational heterogeneity. State-of-the-art predictors struggle to model alternative conformations, fold switches, large-amplitude conformational changes and solution ensembles (Chakravarty *et al.*, 2025 ▸).

The success of *AlphaFold*2 (Jumper *et al.*, 2021 ▸) has stimulated machine-learning approaches focused on inference-time interventions in the model to generate structural diversity. They include enabling or increasing dropout (Raouraoua *et al.*, 2024 ▸; Wallner, 2023 ▸), or manipulating the evolutionary information given as input to the model (Kalakoti & Wallner, 2024 ▸; Wayment-Steele *et al.*, 2024 ▸; Del Alamo *et al.*, 2022 ▸; Stein & Mchaourab, 2022 ▸). Despite promising results on specific families, several studies have emphasized the difficulties in rationalizing the effectiveness of these modifications and interpreting them (Porter *et al.*, 2024 ▸; Bryant & Noé, 2024 ▸). Moreover, these cannot be transferred to protein language model (pLM)-based predictors that do not rely on multiple sequence alignments. Researchers have also actively engaged in the development of deep-learning frameworks based on diffusion, or the more general flow matching, to generate conformational ensembles (Lewis *et al.*, 2025 ▸; Wang *et al.*, 2025 ▸). While being several orders of magnitude cheaper than molecular-dynamics (MD) simulations, these models remain computationally intensive, require massive MD training data and are limited to sampling approximate equilibrium distributions.

This work proposes a new perspective on the protein conformational diversity problem. Instead of learning and sampling from multi-dimensional empirical distributions, we propose to learn eigenspaces (the structure) of the positional covariance matrices in collections of experimental 3D structures and generalize these over different homology levels. The use of experimental structure collections to infer protein dynamics through principal component analysis (PCA) is well established in the literature (Best *et al.*, 2006 ▸; Schneider *et al.*, 2025 ▸; Lombard *et al.*, 2024 ▸; Yang *et al.*, 2009 ▸). The diversity present within – even a modest number of – experimental 3D structures of the same protein or close homologs is a good proxy for the conformational heterogeneity of proteins in solution (Best *et al.*, 2006 ▸) and can generally be (almost fully) explained by a small set of linear vectors, also referred to as modes (Lombard *et al.*, 2024 ▸; Yang *et al.*, 2009 ▸). Moreover, interpolation trajectories performed in PCA space inferred from experimental structures can recapitulate intermediate functional states (Lombard *et al.*, 2024 ▸). Although linear spaces may not be well suited for capturing highly complex nonlinear motions, such as loop deformations, they offer multiple advantages. These include faster learning due to the reduced complexity of the model, improved explainability as the components directly correspond to interpretable data dimensions, faster inference and the straightforward combination or integration of multiple data dimensions.

To summarize, our main contributions are as follow.

(i) We provide a novel formulation of the protein conformational diversity problem.
(ii) We present a novel benchmark representative of the Protein Data Bank (PDB; Berman *et al.*, 2000 ▸) structural diversity compiled with a robust pipeline (Lombard *et al.*, 2024 ▸), along with data- and task-specific metrics.
(iii) We develop an SE(3)-equivariant graph neural network architecture equipped with a novel symmetry-aware loss function for comparing linear subspaces, with invariance to permutation and scaling. Our model, *PETIMOT*, leverages embeddings from pre-trained pLMs, building on prior proof-of-concept work demonstrating that they encode information about functional protein motions (Lombard *et al.*, 2025 ▸).
(iv) *PETIMOT* is trained on sparse experimental data without any use of simulation data, in contrast with *Timewarp*, for instance (Klein *et al.*, 2024 ▸). Moreover, our model does not require physics-based guidance or feedback, unlike that of Wang *et al.* (2025 ▸), for instance.
(v) Our results demonstrate the capability of *PETIMOT*to generalize across protein families (contrary to variational autoencoder-based approaches) and to compare favorably in running time and accuracy to the physics-based normal-mode analysis.

## 2. Related work

### 2.1. Protein structure prediction and generating conformational ensembles

*AlphaFold*2 was the first end-to-end deep neural network to achieve near-experimental accuracy in predicting protein 3D structures, even for challenging cases with low sequence similarity to proteins with resolved structures (Jumper *et al.*, 2021 ▸). Subseqent work has shown that substituting the input alignment by embeddings from a pLM can yield comparable performance (Lin *et al.*, 2023 ▸; Hayes *et al.*, 2024 ▸; Weissenow *et al.*, 2022 ▸; Wu *et al.*, 2022 ▸).

Beyond the single-structure frontier, several studies have underscored the limitations and potential of protein structure predictors (PSP) for generating alternative conformations (Saldaño *et al.*, 2022 ▸; Lane, 2023 ▸; Bryant & Noé, 2024 ▸; Chakravarty *et al.*, 2025 ▸). Approaches focused on repurposing *AlphaFold*2 include dropout-based massive sampling (Raouraoua *et al.*, 2024 ▸; Wallner, 2023 ▸), guiding the predictions with state-annotated templates (Faezov & Dunbrack, 2023 ▸; Heo & Feig, 2022 ▸) and inputting shallow, masked, corrupted, subsampled or clustered alignments (Kalakoti & Wallner, 2024 ▸; Wayment-Steele *et al.*, 2024 ▸; Del Alamo *et al.*, 2022 ▸; Stein & Mchaourab, 2022 ▸). Despite promising results, these approaches remain computationally expensive and their generalizability, interpretability and controllability remain unclear (Bryant & Noé, 2024 ▸; Chakravarty *et al.*, 2025 ▸). More recent work has aimed at overcoming these limitations by directly optimizing PSP learnt embeddings under low-dimensional ensemble constraints (Yu *et al.*, 2025 ▸)

Another line of research has consisted of fine-tuning or re-training *AlphaFold*2 and other single-state PSP under diffusion or flow-matching frameworks (Jing *et al.*, 2024 ▸; Abramson *et al.*, 2024 ▸; Krishna *et al.*, 2024 ▸). More generally, diffusion- and flow matching-based models allow the efficiently generation of diverse conformations conditioned on the presence of ligands or cellular partners (Jing *et al.*, 2023 ▸; Ingraham *et al.*, 2023 ▸; Wang *et al.*, 2025 ▸; Liu *et al.*, 2024 ▸). Despite their strengths, these techniques are prone to hallucination.

The recent availability of large-scale molecular-dynamics (MD) datasets (Vander Meersche *et al.*, 2024 ▸; Siebenmorgen *et al.*, 2024 ▸; Mokhtari *et al.*, 2026 ▸) has opened the door to a parallel line of research training deep-learning models directly on these data to enhance or replace MD exploration. A first group of methods develops machine-learning force fields based on equivariant GNN representations (Wang, He *et al.*, 2024 ▸). A second directly emulates MD trajectories as generative tasks, enabling forward simulation, transition path sampling and trajectory upsampling (Jing, Stärk *et al.*, 2024 ▸; Costa *et al.*, 2024 ▸). A third learns generative models of equilibrium Boltzmann distributions (Noé *et al.*, 2019 ▸; Klein *et al.*, 2024 ▸; Zheng *et al.*, 2024 ▸; Lewis *et al.*, 2025 ▸). For instance, the *BioEmu* model (Lewis *et al.*, 2025 ▸), trained on more than 200 ms of MD simulations and fine-tuned on experimental protein stability measurements, approximates equilibrium conformational distributions at a fraction of the cost of MD simulations, while capturing biologically meaningful conformational changes deposited in the PDB.

### 2.2. Protein conformational heterogeneity manifold learning

Unsupervised, physics-based normal-mode analysis (NMA) has long been effective for inferring functional modes of deformation by leveraging the topology of a single protein 3D structure (Grudinin *et al.*, 2020 ▸; Hoffmann & Grudinin, 2017 ▸; Hayward & Go, 1995 ▸). While appealing for its computational efficiency, the accuracy of NMA strongly depends on the initial topology (Laine & Grudinin, 2021 ▸), limiting its ability to model extensive secondary-structure rearrangements. Recent efforts have sought to address these limitations by directly learning continuous, compact representations of protein motions from sparse experimental 3D structures. These approaches employ dimensionality-reduction techniques, from classical manifold learning methods (Lombard *et al.*, 2024 ▸) to neural network architectures such as variational auto-encoders (Ramaswamy *et al.*, 2021 ▸). By projecting motions onto a learned low-dimensional manifold, these methods enable the reconstruction of accurate, physico-chemically realistic conformations, both within the interpolation regime and near the convex hull of the training data (Lombard *et al.*, 2024 ▸). Additionally, they assist in identifying collective variables from MD simulations, supporting importance-sampling strategies (Chen, Roux *et al.*, 2023 ▸; Belkacemi *et al.*, 2022 ▸; Bonati *et al.*, 2021 ▸; Wang *et al.*, 2020 ▸; Ribeiro *et al.*, 2018 ▸). Despite these advances, such approaches are currently constrained to family-specific models.

### 2.3. SE(3)-equivariant graph neural networks

Graph neural networks (GNNs) have been extensively used to represent protein 3D structures. They are robust to transformations of the Euclidean group, namely rotations, reflections and translations, as well as to permutations. In their simplest formulation, each node represents an atom and any pair of atoms are connected by an edge if their distance is smaller than a cutoff or among the smallest *k* interatomic distances. Many studies have proposed to enrich this graph representation with SE(3)-equivariant features informing the model about interatomic directions and orientations (Ingraham *et al.*, 2019 ▸; Jing *et al.*, 2020 ▸; Dauparas *et al.*, 2022 ▸; Krapp *et al.*, 2023 ▸; Wang, Wang *et al.*, 2024 ▸). To go beyond local 3D neighborhoods while maintaining subquadratic complexity, *Chroma* adds in randomly sampled long-range connections (Ingraham *et al.*, 2023 ▸).

## 3. Data representation and problem formulation

To generate training data, we exploit experimental protein single-chain structures available in the PDB. We first clustered these chains based on their sequence similarity. Then, within each cluster, we aligned the protein sequences and used the resulting mapping to superimpose the 3D coordinates (Lombard *et al.*, 2024 ▸). It may happen that some residues in the multiple sequence alignment do not have resolved 3D coordinates in all conformations. To account for this uncertainty, we assigned a confidence score *w*_*i*_ to each residue *i* computed as the proportion of conformations including this residue. The 3D superimposition sets the conformations’ centers of mass to zero and then aims to determine the optimal least-squares rotation minimizing the root-mean-square deviation (r.m.s.d.) between any conformation and a reference conformation, while accounting for the confidence scores (Kabsch, 1976 ▸; Kearsley, 1989 ▸), 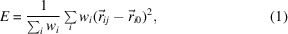
where 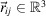 is the *i*th centered coordinate of the *j*th conformation and 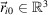 is the *i*th centered coordinate of the reference conformation. Next, we defined our ground-truth targets as eigenspaces of the coverage-weighted C^α^-atom positional covariance matrix, 
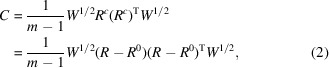
where *R* is the 3*N* × *m* positional matrix, with *N* the number of residues and *m* the number of conformations, *R*^0^ contains the coordinates of the reference conformation and *W* is the 3*N* × 3*N* diagonal coverage matrix. The covariance matrix is a 3*N* × 3*N* square matrix, symmetric and real. We decompose *C* as *C* = *YDY*^T^, where *Y* is a 3*N* × 3*N* matrix with each column defining a coverage-weighted eigenvector or a principal component that we interpret as a *linear motion*. *D* is a diagonal matrix containing the eigen­values. The latter highly depend on the sampling biases in the PDB and thus we do not aim to predict them.

### 3.1. Problem formulation

For a protein of length *N*, let *Y* be 3*N* × *Korthogonal* ground-truth deformations, 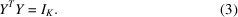
Our goal is to find coverage-weighted vectors 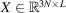 whose components *l approximate* some components *k* of the ground truth *Y*: 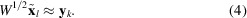
Below, we provide three alternative formulations for this problem. *PETIMOT*’s loss function serves two key purposes: it enables effective training of the network to predict subspaces representing multiple distinct modes of deformations, *i.e.* with low overlap between the subspace’s individual linear vectors, while preventing convergence to a single dominant mode.

### 3.2. The least-square formulation

To evaluate a predicted motion direction against a ground-truth direction, we use a least-square (LS) error, which, together with mean absolute error (MAE), is among the most accepted metrics for regression tasks. Here, we have specifically adapted it to the challenge of evaluating directional motion vectors rather than static coordinates, and scaled between 0 and 1 for better training, interpretability and usability.

For each protein of length *N* with a coverage *W*, we compute the weighted pairwise *least-square difference*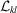 between ground-truth directions *Y* and predicted motion directions *X* for each pair of a *k* direction in the ground truth and an *l* direction in the prediction as
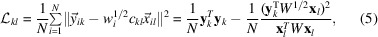
where we scaled the ground-truth tensors such that *Y*^T^*Y* = *NI*_*K*_ and we used the fact that the optimal scaling coefficients *c*_*kl*_ between the *k*th ground-truth vector and the *l*th prediction are given by 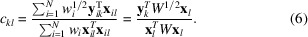
This invariance to global scaling is motivated by the fact that we aim to capture the relative magnitudes and directions of the motion patterns rather than their sign or absolute amplitudes.

### 3.3. Linear assignment problem

We then formulate an *optimal linear assignment problem*to find the minimum-cost matching between the ground-truth and the predicted directions. Specifically, we aim to solve the following assignment problem for the least-square (LS) costs, 
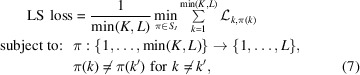
where *K* and *L* are the number of ground-truth and predicted directions, respectively, and π(*k*) represents the index of the predicted direction assigned to the *k*th ground-truth direction. This formulation ensures an optimal one-to-one matching, while accommodating cases where the number of predicted and ground-truth directions differs. We backpropagate the loss only through the optimally matched pairs, using *SciPy*linear_sum_assignment. We have also tested a smooth version of the loss above with continuous gradients, but it did not improve the performance.

### 3.4. The subspace-coverage formulation

We propose another formulation of the problem in terms of the subspace-coverage metrics (Amadei *et al.*, 1999 ▸; Leo-Macias *et al.*, 2005 ▸; David & Jacobs, 2011 ▸). Specifically, we sum up *squared sinus* (SS) dissimilarities between ground-truth and predicted directions (formally computed as one minus squared cosine similarity), 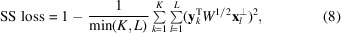
where the subspace 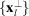 is obtained by orthogonalizing the coverage-weighted predicted linear subspace 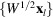, where 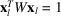, using the Gram–Schmidt process. This operation ensures that the loss ranges from zero for identical subspaces to one for mutually orthogonal subspaces and avoids artificially inflating the SS loss due to redundancy in the predicted motions. The order in which the predicted vectors are orthogonalized does not influence the loss, guaranteeing stable training. Appendix *A*[App appa] proves this statement. The SS loss is conceptually similar to the comparison of angles between subspaces: see a few recent examples of such subspace comparison from other ML domains (Zhu *et al.*, 2021 ▸; Feng *et al.*, 2023 ▸; Chen, Miao *et al.*, 2023 ▸; Hawke *et al.*, 2024 ▸; Schlaginhaufen & Kamgarpour, 2024 ▸).

### 3.5. Independent subspace (IS) loss

We can substitute the orthogonalization procedure by using an auxiliary loss component to maximize the rank of the predicted subspace. For this purpose, we chose the squared cosine similarity computed between pairs of predicted vectors. The final expression for the *independent subspace* (IS) loss is 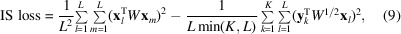
where the predictions {**x**_*l*_} are normalized prior to the loss computation such that 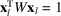 and the scaling factors ensure that the loss ranges between 0 and 1. Appendix *A*[App appa] analyses the stability of this formulation.

## 4. Architecture

**Figure 1:**
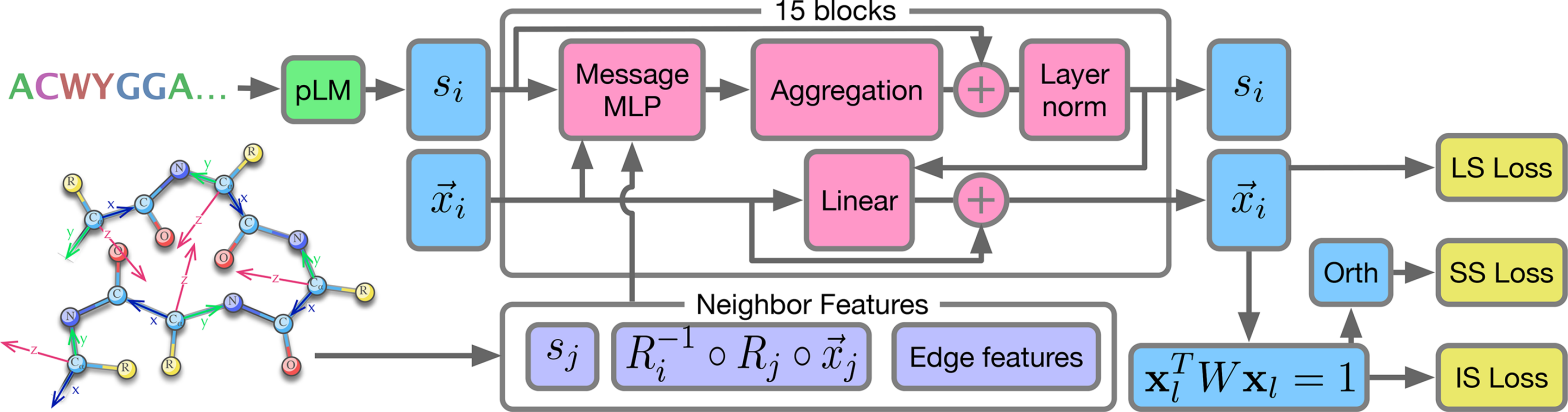
Overview of *PETIMOT* architecture. The model processes sequence embeddings (*s*) and motion vectors (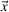) through 15 message-passing blocks. The GNN topology and edge features are defined from the input 3D coordinates. Edge features encode 3D geometrical properties such as the relative spatial relationships between residue pairs. Each block updates both *s* and 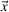 representations by aggregating information from neighboring residues. SE(3) equivariance is achieved by computing the features of neighbors *j* in the reference frame of the central residue *i*. Three types of losses (LS, SS and IS) are computed, with prior normalization of the predictions for the IS and SS losses, and an additional orthogonalization of the predictions for the SS loss.

We solve the problem formulated above with a pLM-informed SE(3)-equivariant graph neural network called *PETIMOT* (Fig. 1 ▸). *PETIMOT* takes as input a protein sequence of length *N*, converted into an embedding **s** by a pre-trained pLM, along with 3D coordinates, and outputs a set of linear motions 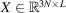.

### 4.1. Dual-track representation

*PETIMOT* processes protein sequences through a message-passing neural network that simultaneously handles residue embeddings and motion vectors in local coordinate frames (Fig. 1 ▸). For each residue *i*, we define and update a node embedding 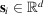 initialized from pLM features and a set of *K* motion vectors 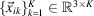 initialized randomly. The message-passing procedure is detailed in Algorithm B1 in Section B2 (Appendix *B*[App appb]). The protein is represented as a graph where nodes correspond to the residues and edges capture spatial relationships. We connect each residue *i* to its *k* nearest neighbors based on C^α^ distances in the input structure and *l* randomly selected residues. This hybrid connectivity scheme ensures both local geometric consistency and global information flow, while maintaining sparsity for computational efficiency. Indeed, our model scales *linearly* with the length *N* of a protein. In our base model we set *k* = 5 and *l* = 10.

### 4.2. Node and edge features

We chose *ProstT*5 as our default pLM for initializing node embeddings (Heinzinger *et al.*, 2024 ▸). This structure-aware pLM offers an excellent balance between model size – including the number of parameters and embedding dimensionality – and performance (Lombard *et al.*, 2025 ▸). Each residue’s backbone atoms (N, CA, C) define a local reference frame through a rigid transformation *T*_*i*_ ∈ SE(3). For each residue pair (*i*, *j*), we compute their relative transformation 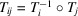, from which we extract the rotation *R*_*ij*_ ∈ SO(3) and translation 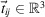. Under global rotations and translations of the protein, these relative transformations remain invariant. Edge features *e*_*ij*_ provide an SE(3)-invariant encoding of the protein structure through relative orientations, translational offsets, protein chain distance and a complete description of peptide-plane positioning captured by pairwise backbone-atom distances. See Section B2 for further details. Licenses for used resources are listed in Appendix *D*[App appd].

## 5. Results

### 5.1. Training and evaluation

We trained *PETIMOT* against linear motions extracted from all ∼750 000 protein chains in the PDB (as of June 2023) clustered at 80% sequence identity and coverage. Our full training data comprises 7335 conformational collections, which we augmented by computing the motions with respect to five reference conformations per collection. As a result, the full training set comprises 36 675 samples. This reference dataset encompasses conformations solved by multiple experimental techniques, including 56 866 cryo-EM structures (30.5%) and 2187 NMR structures (about 1.5%). This ensures the representation of diverse conformational states beyond those accessible to X-ray crystallography. Moreover, only 5.6% of the training samples exhibit a maximum pairwise r.m.s.d. below 2 Å, indicating that the vast majority of the dataset captures substantial conformational diversity. We set the numbers of predicted and ground-truth motions, *K* = *L* = 4. See Sections B1 and B4 for further details. At inference, we consider *w*_*i*_ = 1, ∀*i* = 1 … *N*. We rely on four main evaluation metrics aimed at addressing the following questions.

**Figure 2:**
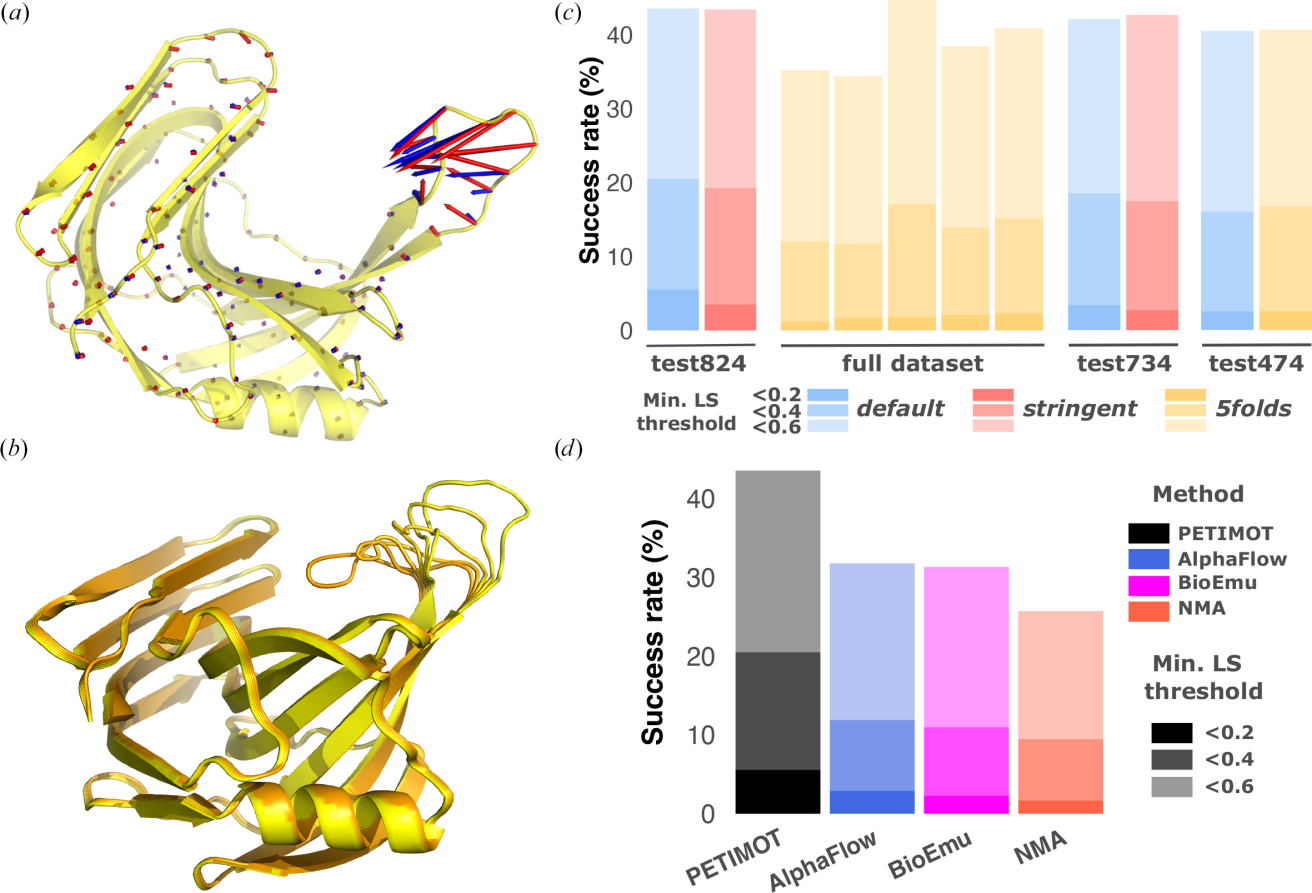
*PETIMOT* prediction visualization, evaluation and comparison with other methods. (*a*, *b*) Prediction for *B. subtilis* xylanase A (PDB entry 3exu, chain *A*). (*a*) The predicted and ground-truth vectors are in blue and red, respectively (LS = 0.15). (*b*) Trajectory generated by deforming the protein structure along the predicted motion. (*c*, *d*) Success rates computed as the proportions of test proteins with mimimum LS below 0.2 (dark), 0.4 (mild) and 0.6 (light). (*c*) Comparison of *PETIMOT*’s *default* (blue), *stringent* (tomato red) and *5folds* (gold) models. Test sets avoid data leakage at different levels. Test824: less than 80% sequence similarity to any collection used for training. Full dataset: folds are defined based on strict sequence and structural similarity filters. Test734: less than 30% sequence similarity to training collections. Test474: less than 30% sequence similarity and no significant structural similarity to training collections. (*d*) Comparison of *PETIMOT*’s *default* model (black) with *AlphaFlow* (blue), *BioEmu* (magenta) and the NMA (tomato red) on test824.

(i) Is *PETIMOT* able to approximate at least one of the main linear motions of a given protein? For this, we rely on the minimum LS error over all possible pairs of predicted and ground-truth vectors. A prediction with LS ≤ 0.2 almost perfectly superimposes with the ground-truth motion (Figs. 2 ▸*a* and 2 ▸*b*). We consider predictions with LS > 0.6 as inaccurate as they typically miss or indicate completely wrong directions for a large part of the residues involved in the motion. By comparison, the LS errors computed for random predictions are typically above 0.9.
(ii) To what extent does *PETIMOT* capture the main motion linear subspace of a given protein? For this, we use the global SS error.
(iii) Is *PETIMOT* able to identify the residues that move the most? Here, we rely on the magnitude error, 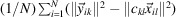.
(iv) Can *PETIMOT* be used to generate conformations resembling experimentally resolved functional protein states? For this, we generate conformations by deforming the input protein structure along the predicted motions and compute their r.m.s.d. to five diverse conformations selected from the ground-truth collection. See Section B1 for further details.

### 5.2. Robustness and generalization

We tested *PETIMOT*’s generalization capabilities using three training protocols. In two of them, we randomly split the reference dataset into 70% for training, 15% for validation and 15% for test, where any test protein has less than 80% (*default*) or 30% (*stringent*) sequence similarity to the training proteins. In addition, we conducted fivefold cross-validation ensuring that each fold’s training set did not contain any protein chain sharing significant structural or sequence similarity with the test set (*5folds*). This protocol strictly prevents data leakage and provides robust evaluation across our complete dataset (Section B1). *PETIMOT*’s performance is robust and generalizable across the different data partitions, with success rates, defined as the fractions of test proteins with minimum LS ≤ 0.6, in the 35–45% range (Fig. 2 ▸*c*, Tables 2 and 3). Moreover, it showed limited sensitivity to the choice of reference conformation, predicting acceptable motions for at least two out of five reference conformations in 80% of successful collections (Fig. 10*a*). See Appendix *C*[App appc] for further details.

### 5.3. Biological relevance

To assess the biological relevance of *PETIMOT*’s predictions, we focused on three case studies: open–closed transitions, fold switches and multi-state cryo-EM resolved structures. For open–closed transitions, we considered the well established iMod benchmark (Lopéz-Blanco *et al.*, 2011 ▸) comprising a couple of tens of proteins with a wide variety of motions (hinge, shear, allosteric and complex motions) often associated with ligand or partner binding. *PETIMOT-5folds* predicted these transitions with high accuracy, achieving a 86% success rate with an average minimum LS error of 0.41 ± 0.18 and an average minimum magnitude error of 0.14 ± 0.07. For fold switches, we compiled a dataset of six metamorphic proteins from Wayment-Steele *et al.* (2024 ▸). *PETIMOT-5folds* achieved a success rate of 37% on these challenging cases, with a minimum LS error of 0.67 ± 0.17 and a minimum magnitude error of 0.25 ± 0.14. Our approach performed particularly well on KaiB, as also highlighted in Wayment-Steele *et al.* (2024 ▸). The minimum LS error is 0.45 starting from the ground state (PDB entry 2qke, chain *C*) and 0.57 starting from the FS state (PDB entry 5jyt, chain *A*). Finally, we considered the ATPase NSF, whose experimental structures correspond to ATP/ADP-bound states, and 20S supercomplex conformations from cryo-EM studies (Zhao *et al.*, 2015 ▸; White *et al.*, 2018 ▸). The functionally relevant motions involve large-amplitude rigid-body domain movements and loop rearrangements. The first linear PCA mode explains 57% of the variance and four modes are required to explain 90%. *PETIMOT* successfully captures this complex motion subspace with a minimum LS error as low as 0.32 and a global SS error of 0.30, demonstrating its ability to predict not just single motions but biologically meaningful motion subspaces.

### 5.4. Comparison with the physics-based unsupervised NMA

**Figure 3:**
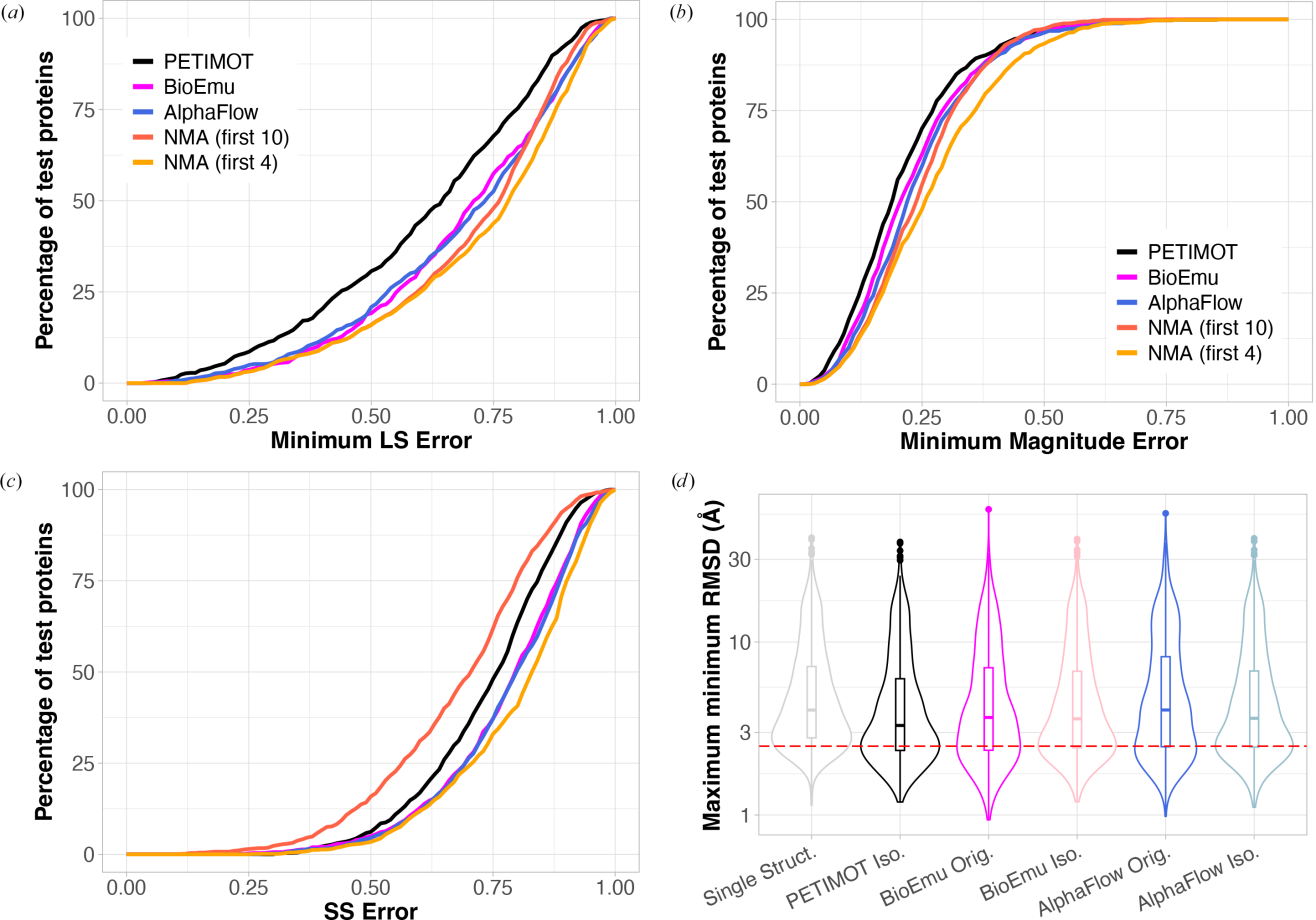
*PETIMOT*’s performance on the test set and comparison with other methods. *PETIMOT-default* is compared with *AlphaFlow*, *BioEmu* and the NMA on test824. For each protein, we evaluate the four motions predicted by *PETIMOT* or inferred from *AlphaFlow*/*BioEmu*-predicted ensembles against the four ground-truth motions. We allow the NMA more flexibility by considering the ten lowest frequency modes. (*a*) Minimum LS error, computed for the best-matching pair of predicted and ground-truth motions. (*b*) Minimum magnitude error. (*c*) Global SS error, reflecting ground-truth subspace coverage. (*d*) Distributions of maximum minimum r.m.s.d.: within each generated ensemble, we identify the conformations closest to the reference structures and compute the maximum r.m.s.d. among them. If this value is below 2.5 Å then we consider that all reference structures are covered by the ensemble. Single Struct. refers to the input structure given to *PETIMOT*. Orig. stands for the original ensembles generated directly from *BioEmu* and *AlphaFlow*, while Iso. stands for isotropic ensembles generated from motions predicted by *PETIMOT* or inferred from *BioEmu*/*AlphaFlow* original ensembles.

We primarily compare *PETIMOT* with the NMA, a cost-effective approach for predicting the motion directions energetically accessible to a protein 3D structure. We considered the ten normal modes with the lowest frequencies. This asymmetrical evaluation, compared with *PETIMOT*’s four predicted motions, ensures a strong baseline and follows the suggestion that weighted mixtures of low-frequency modes can be more informative than individual modes (Kolossváry, 2024 ▸). *PETIMOT* produced acceptable predictions for almost 40% of the dataset, while the NMA’s success rate is 25% (Fig. 2 ▸*d*, Tables 2 and 3), and *PETIMOT* achieved lower errors than the NMA in two thirds of the proteins (Fig. 3 ▸*a*). While *PETIMOT*’s individual predictions better match the ground-truth motions, the ten-mode subspace predicted by the NMA has higher overlap with the four-mode ground-truth subspace than the four-mode *PETIMOT* subspace (Fig. 3 ▸*c* and Table 3; global SS error of 0.67 ± 0.16 versus 0.73 ± 0.14). This suggests that low-frequency NMA modes collectively span the conformational subspace well, albeit at the cost of losing individual mode precision. When restricting to the first four normal modes, the global SS error drastically increases to 0.79 ± 0.14 (Fig. 3 ▸*c*). These results hold when varying the density and resolution of the elastic network model (ENM; see Tables 2 and 4): among C^α^ ENM variants, a cutoff of 10 Å yields the best performance, and switching to an all-atom representation provides only marginal gains (success rate 28.52% versus 25.73%), leaving *PETIMOT*’s advantage intact across all configurations. Moreover, *PETIMOT* outperformed the NMA in terms of consistency across conformations (Fig. 10*b*) and was 2.75 times faster at inference (Table 3).

### 5.5. Comparison with generative models

We considered the flow-matching or diffusion-based generative models *AlphaFlow* and *BioEmu* as additional baselines. *AlphaFlow* was trained solely on the PDB, while *BioEmu* was trained on massive amounts of experimental structures, 3D models and MD conformations. To ensure a fair comparison, we relied on both our motion-specific metrics and on commonly used metrics for comparing conformational ensembles (see Section B5). We acknowledge a potential data leakage between *AlphaFlow* and *BioEmu* training data and our test set. *PETIMOT* outperforms both ensemble-based methods on motion subspace metrics (Figs. 3 ▸*a*–3 ▸*c*), with a 43.57% success rate versus ∼31% for *AlphaFlow* and *BioEmu* (Fig. 2 ▸*d*), and a substantially lower global SS error (Fig. 3 ▸*c*and Table 3; 0.73 versus 0.77–0.78). Cases where *PETIMOT* produces highly inaccurate predictions (minimum LS loss above 0.7) while the baselines are clearly successful (minimum LS loss below 0.4) are extremely limited (less than five per baseline; see, for instance, Fig. 15). See Appendix *C*[App appc] for further details.

Beyond predicting linear motions, *PETIMOT* allows the straightforward generation of conformational ensembles or trajectories by deforming the input protein 3D structure. We showcase this functionality on xylanase A from *Bacillus subtilis* (Figs. 2 ▸*a* and 2 ▸*b*). We used *PETIMOT*’s best predicted motion to generate physically realistic conformations representing the open-to-closed transition of the xylanase A thumb. More broadly, the conformational ensembles generated by randomly sampling *PETIMOT*’s predicted motions yield an r.m.s.f. Pearson correlation of 0.59 ± 0.23 against ensembles derived from ground-truth motions (Table 1 ▸), outperforming *AlphaFlow* (0.51 ± 0.25) and *BioEmu* (0.52 ± 0.287). When assessed against representative experimental structures, *PETIMOT* ensembles achieve higher or similar coverage compared with the baselines (Table 1 ▸ and Fig. 3 ▸*d*). All representatives are approximated with r.m.s. deviations lower than 2.5 Å in 29.96% of the cases. This is 13 percentage points higher than when considering only the input structure alone, which we used as a trivial lower bound for *PETIMOT*. By comparison, *BioEmu*’s original ensembles cover 28.90%, confirming its ability to produce diverse and biologically relevant conformations. The average minimum r.m.s.d. to experimental structures tells a consistent story, with *PETIMOT*’s isotropic sampling achieving the lowest value (Table 1 ▸, 3.46 ± 2.83 Å). These results demonstrate that the quality of *PETIMOT*’s predicted motion subspace – as measured by our specialized metrics – directly translates to competitive conformational flexibility and coverage, even when using a simple isotropic sampling protocol.

### 5.6. Comparison of problem formulations

Our base model combining the LS and SS losses with equal weights outperforms all three individual losses, LS, SS and IS (Figs. 6 and 7). It strikes an excellent balance between approximating individual motions with high accuracy (Fig. 6*a*) and globally covering the motion subspaces (Fig. 6*b*). By comparison, the SS and IS losses tend to underperform on individual motions, while the LS loss tends to provide lower coverage of the ground-truth subspaces. See Section B6 for further details.

### 5.7. Contribution of sequence and structure features

**Figure 5:**
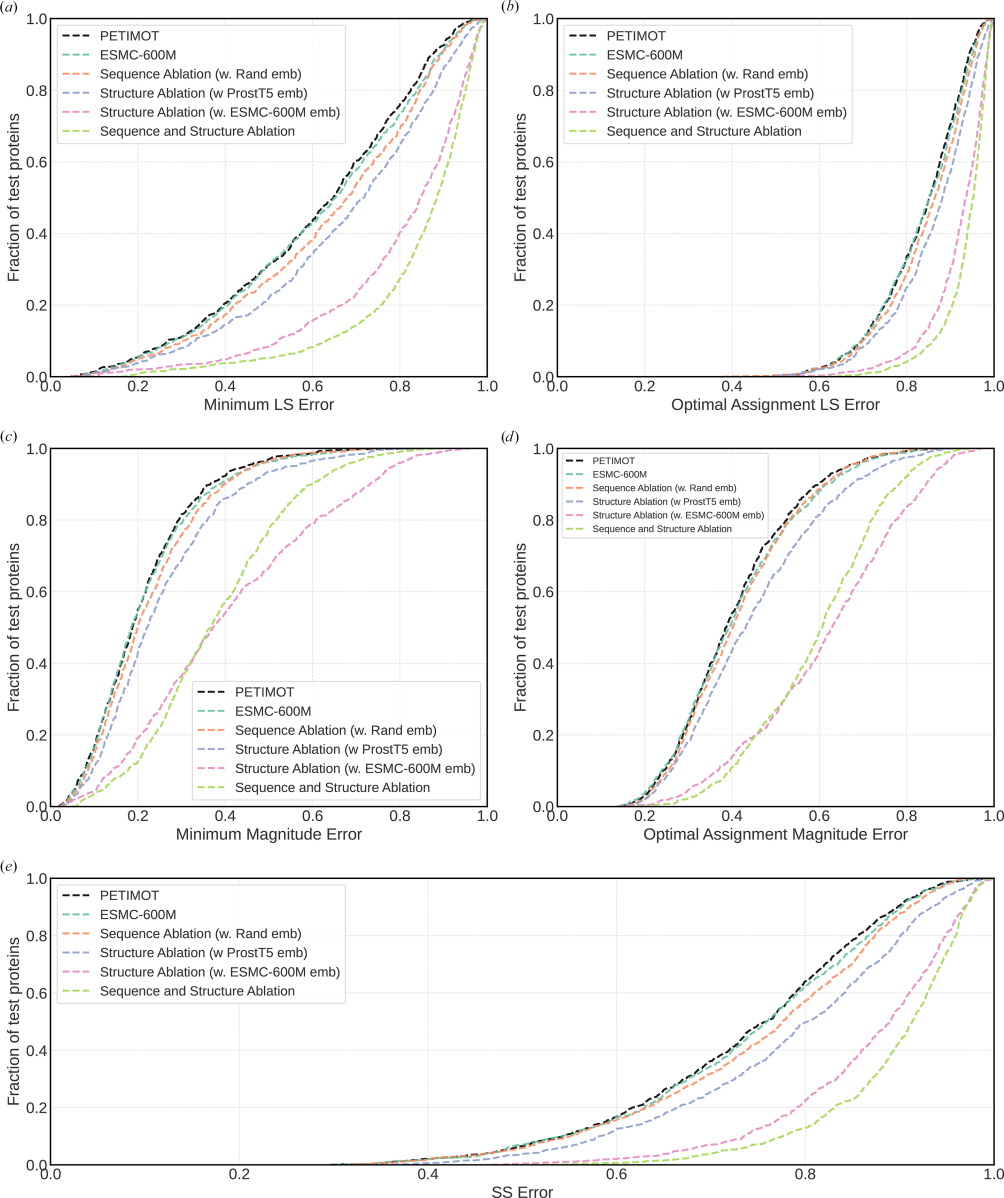
Structure and sequence information ablation study. We report cumulative curves for LS error (*a*, *b*), magnitude error (*c*, *d*) and SS error (*e*). For each protein, we computed the LS and magnitude errors either for the best-matching pair of predicted and ground-truth vectors (*a*, *c*) or for the best combination of four pairs of predicted and ground-truth vectors (*b*, *d*).

**Figure 6:**
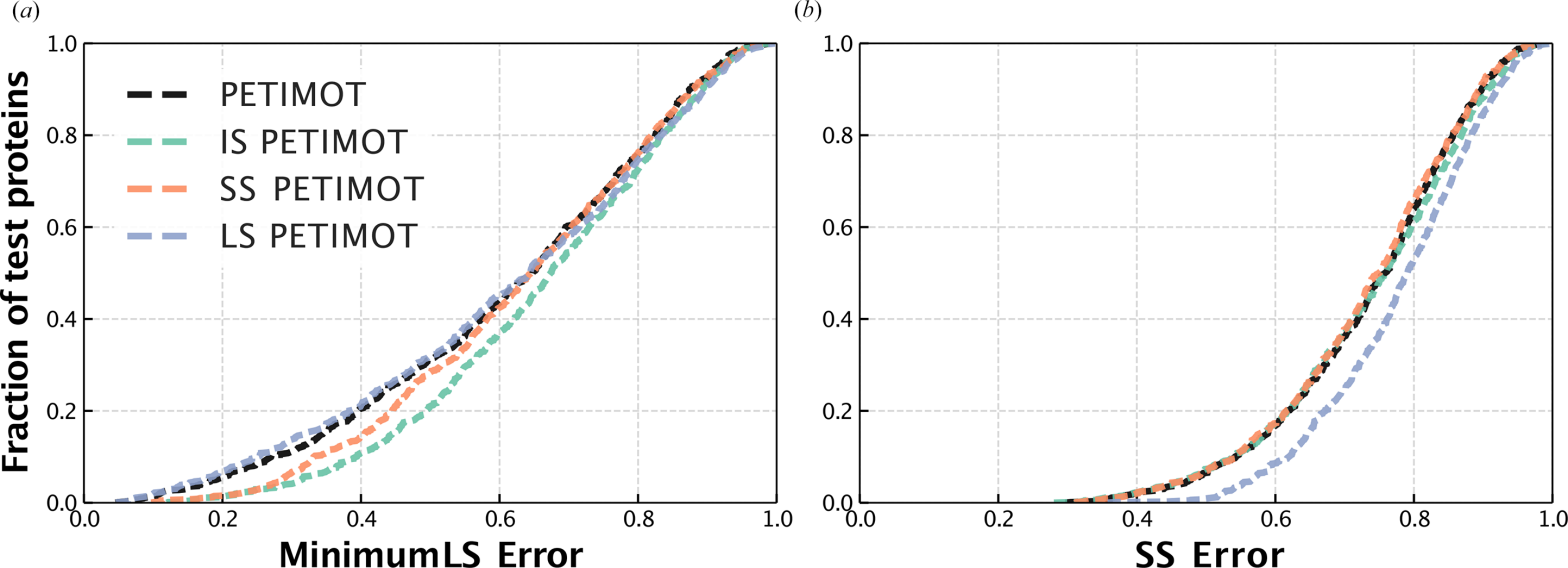
Performance comparison of different problem formulations. We report the cumulative curve for the minimum LS error (*a*) (best-matching pair) and the global SS error (*b*) computed over the test set. The loss of the base model is LS + SS.

**Figure 7:**
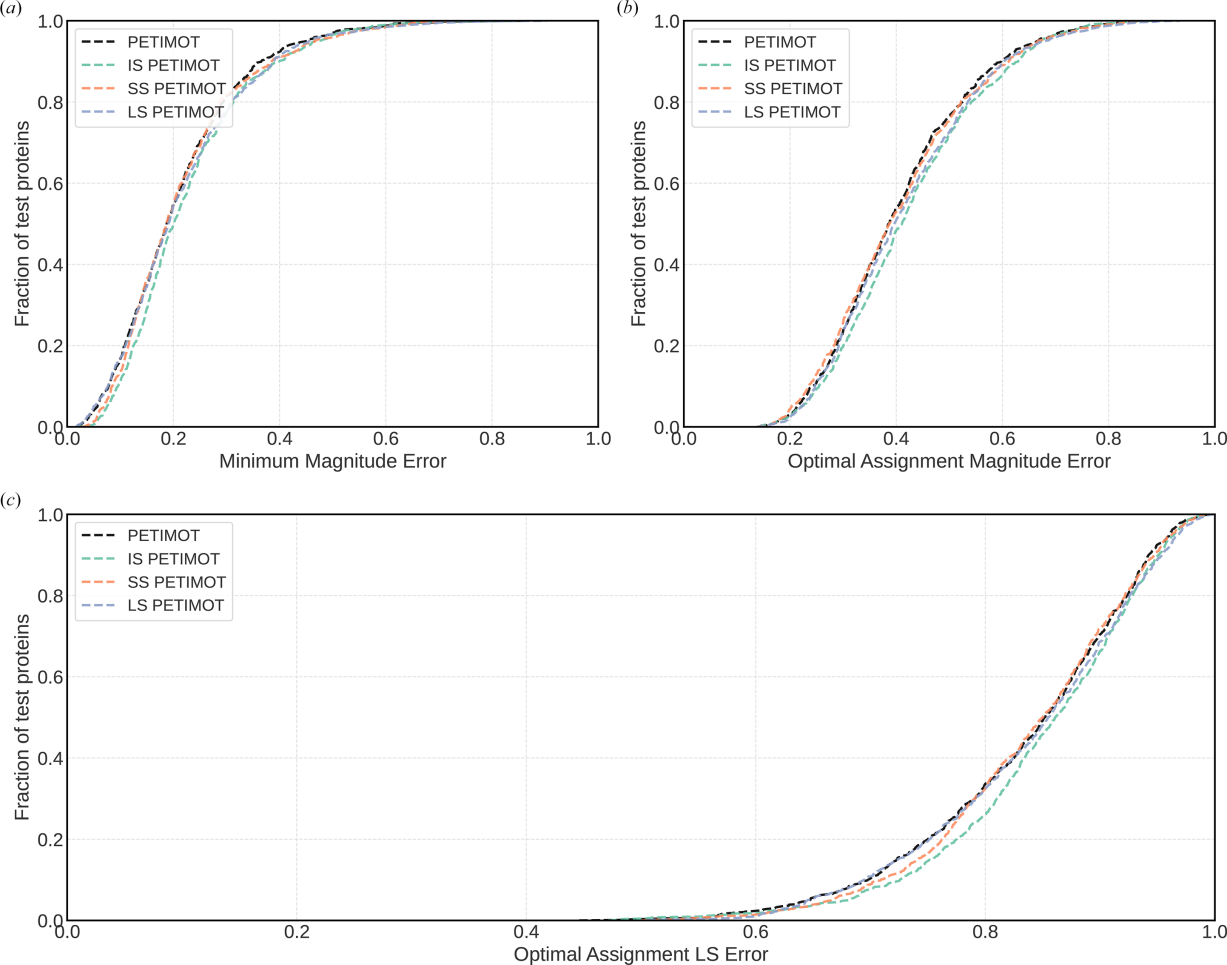
Performance comparison of different problem formulations. We report cumulative curves for magnitude error, corresponding to the best-matching pair of predicted and ground-truth vectors (*a*) (Min.) or the best combination of four pairs of predicted and ground-truth vectors (*b*) (OLA) and OLA LS error (*c*).

**Figure 8:**
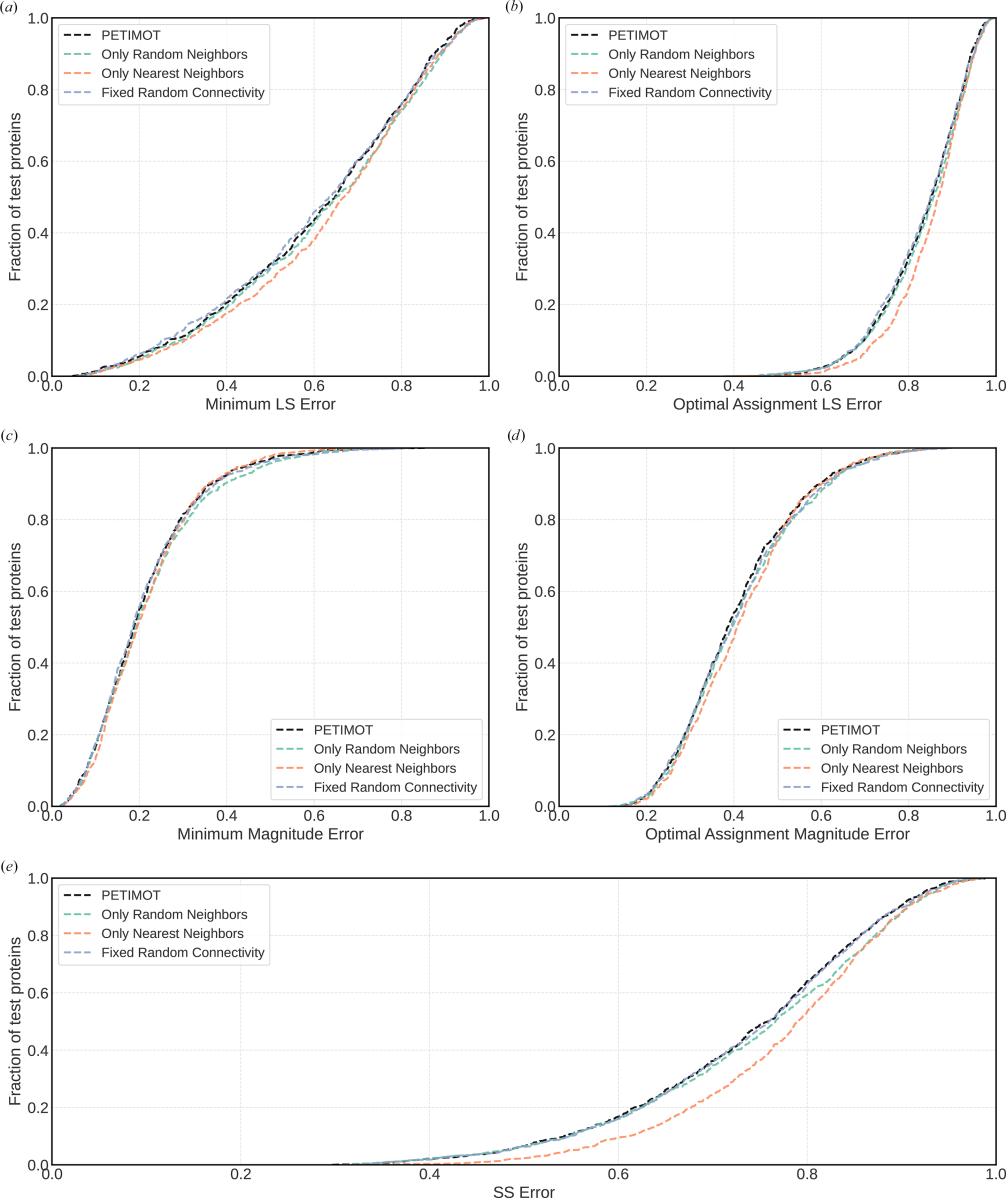
Graph-connectivity ablation. We report cumulative curves for LS error (*a*, *b*), magnitude error (*c*, *d*) and SS error (*e*). For each protein, we computed the error either for the best-matching pair of predicted and ground-truth vectors (*a*, *c*) or for the best combination of four pairs of predicted and ground-truth vectors (*b*, *d*). Only random neighbors: each residue (node) is connected to 15 randomly chosen residues and the connectivity changes after each layer. Only nearest neighbors: each residue (node) is connected to its 15 nearest neighbors in the input 3D structure. Fixed random connectivity: each residue (node) is connected to 15 residues randomly chosen at the beginning.

We performed an ablation study to assess the contribution of sequence and structure information to our architecture. Our results show that *ProstT*5 slightly outperforms the more recent and larger pLM *ESM-Cambrian* 600M (*ESM-C*; ESM Team, 2024 ▸; Fig. 5). Geometrical information about protein structure provides the most significant contribution, as replacing *ProstT*5 embeddings with random numbers has only a small impact on network performance. Conversely, the network’s performance without structural information strongly depends on the chosen pLM. While the structure-aware embeddings from *ProstT*5 partially compensate for missing 3D structure information, relying solely on *ESM-C* embeddings results in poor performance (Fig. 5). Moreover, connecting each residue to its 15 nearest neighbors (sorted according to C^α^–C^α^ distances) in the protein graph results in lower performance compared with introducing randomly chosen edges or even fully relying on random connectivity (Fig. 8). See Section B6 for further details.

### 5.8. Generalization to MD data

**Figure 9:**
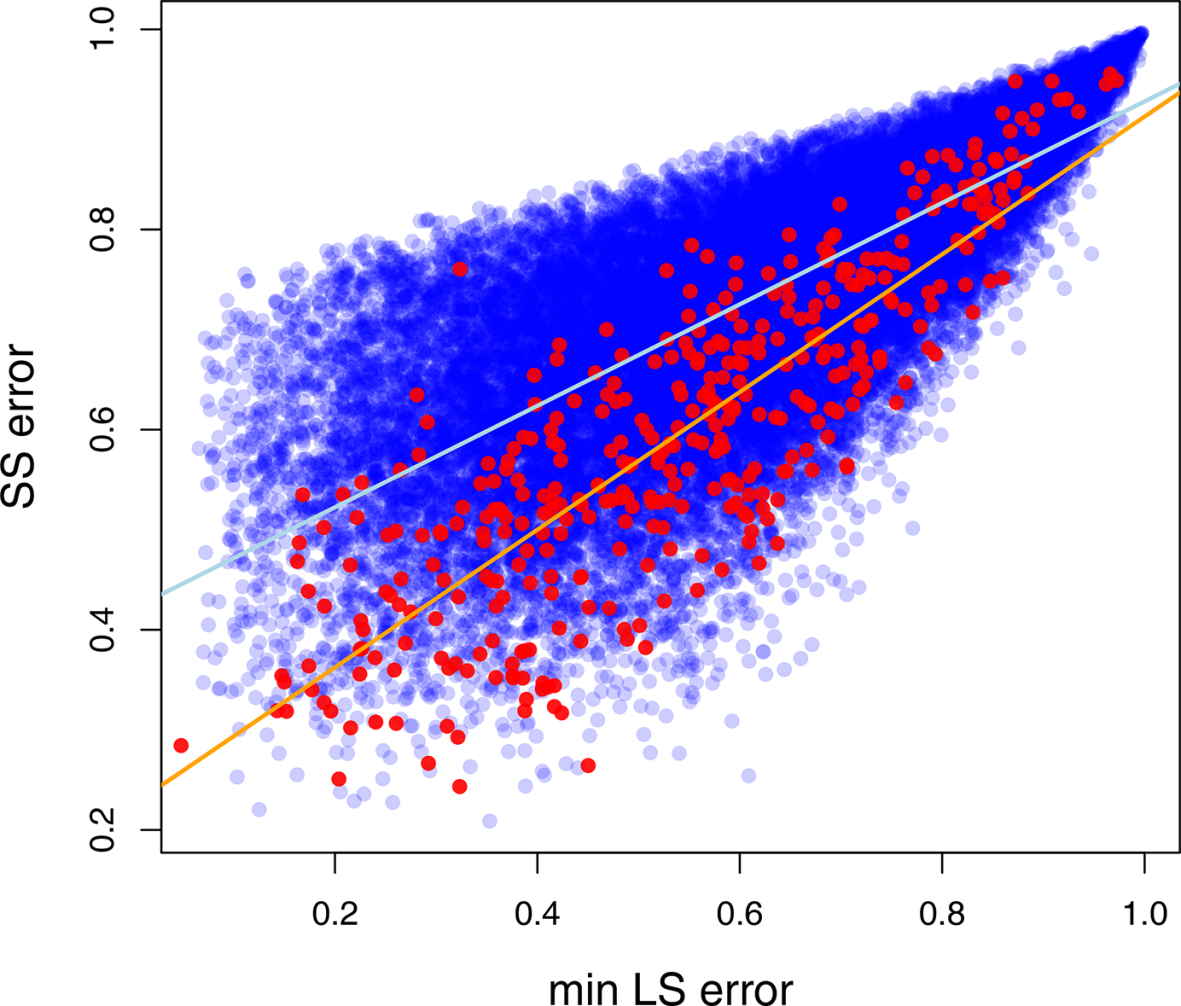
SS error as a function of minimum LS error. Blue dots are samples from our PDB dataset, while red dots correspond to the ATLAS MD samples.

**Figure 10:**
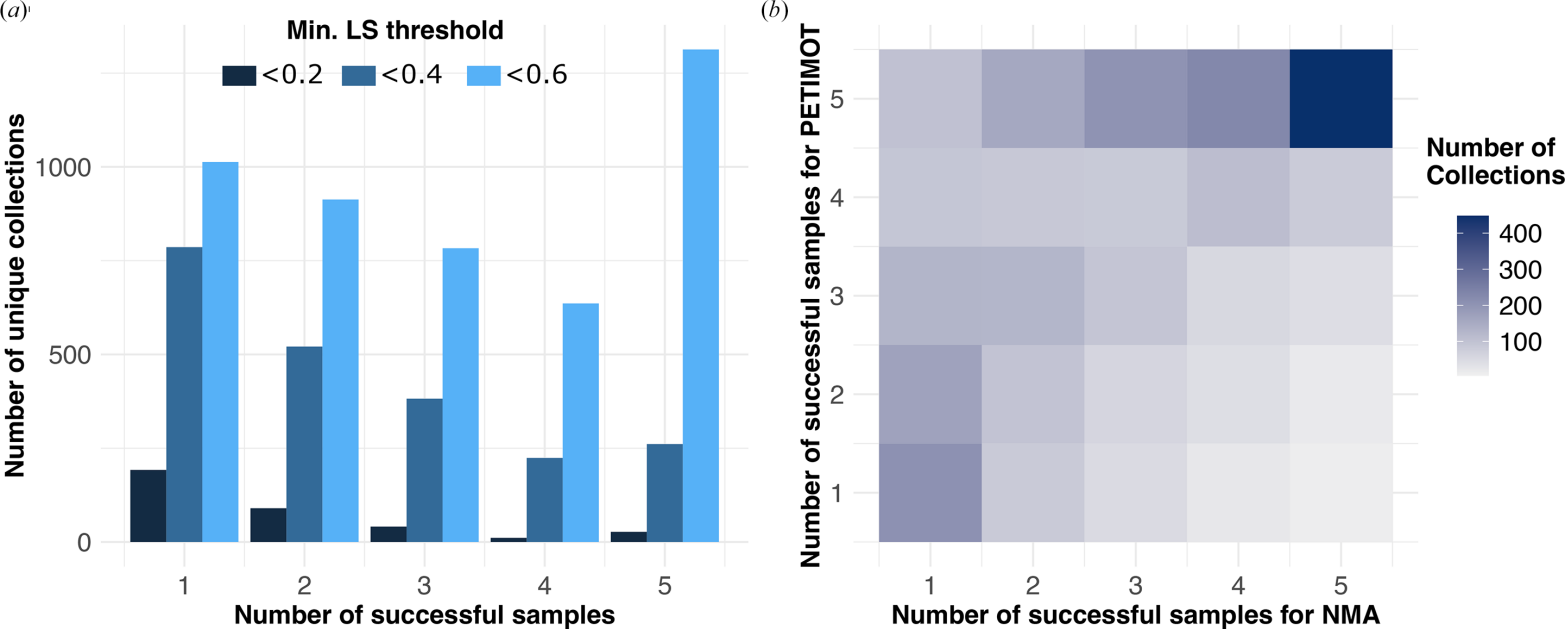
Influence of the reference conformation on *PETIMOT* performance. (*a*) Number of samples per conformational collection for which *PETIMOT-5folds* predicted at least one acceptable motion, with LS below 0.2 (dark), 0.4 (mild) or 0.6 (light). The numbers of successful collections are 361 (4.92% of 7335 in total), 2174 (29.64%) and 4658 (63.50%), respectively. (*b*) Heatmap comparing the number of successful samples, with minimum LS below 0.6, for *PETIMOT-5folds* (*y* axis) and the NMA (*x* axis). The number of successful collections for both methods is 2822 (38.47% of 7335 in total).

**Figure 15:**
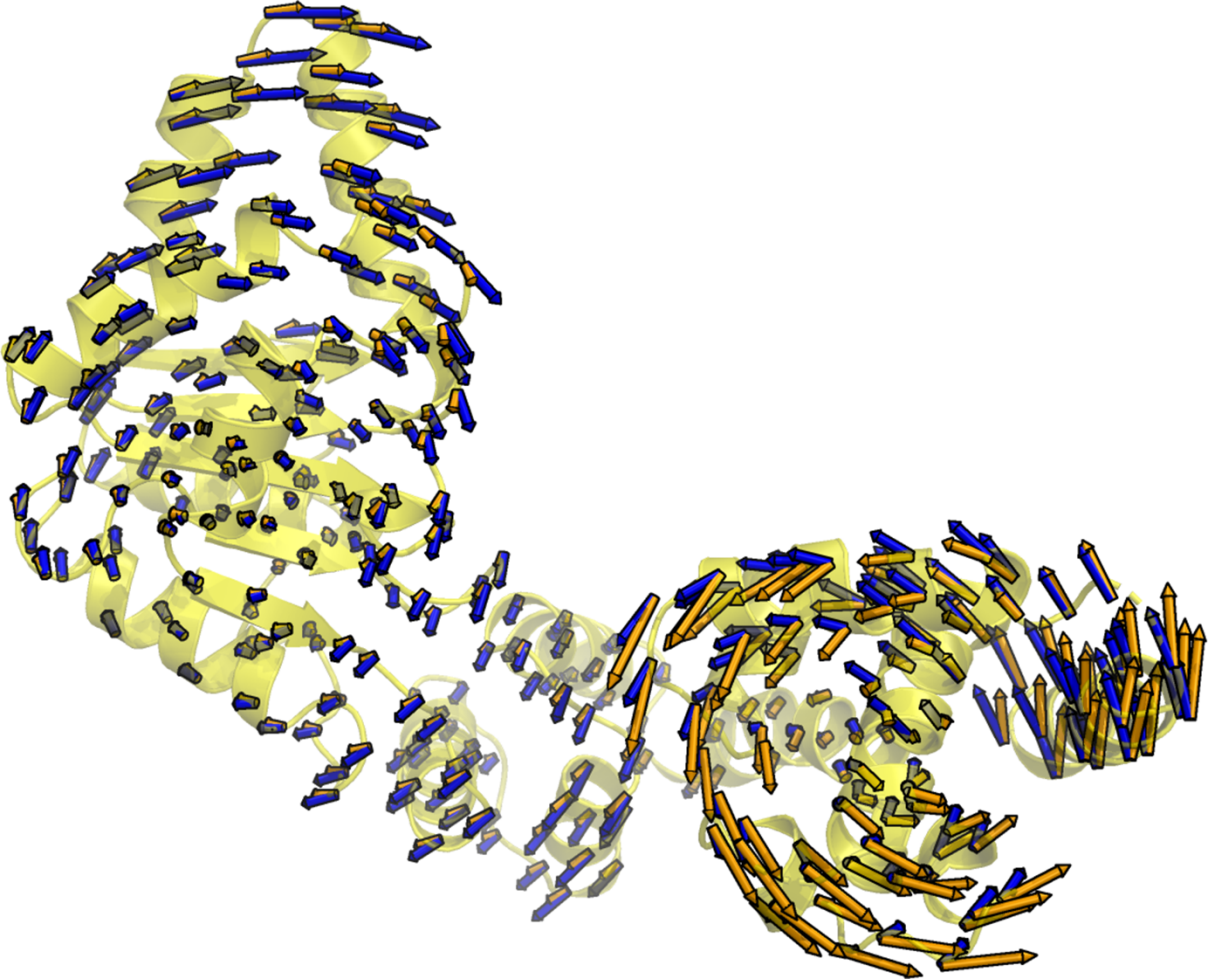
Visualization of predicted motion vectors. *PETIMOT* is in blue (minimum LS = 0.73) and the NMA is in orange (minimum LS = 0.30) for PDB entry 2hcb (chain *C*).

To further assess *PETIMOT*’s robustness, we evaluated it on MD trajectories from the ATLAS dataset (Vander Meersche *et al.*, 2024 ▸). We identified 400 protein chains common to both the ATLAS set and our dataset, providing an independent MD benchmark (see Section B7). To ensure rigorous evaluation without data leakage, for each ATLAS protein chain we used the corresponding *PETIMOT-5folds* model trained on the fold where that specific chain was held out from training (ensuring no training exposure). *PETIMOT-5folds* achieved a 60% success rate on this MD data, with a minimum LS error of 0.55 ± 0.19, a minimum magnitude error of 0.17 ± 0.11 and a global SS error of 0.60 ± 0.16. These performance metrics are significantly better than those obtained on experimental structures. Moreover, the association between minimum LS error and SS error is higher, with an adjusted *R*^2^ of 0.71 versus 0.60 on the PDB dataset (Fig. 9). These results demonstrate that *PETIMOT* generalizes to MD data without re-training or fine-tuning.

### 5.9. Limitations

*PETIMOT*’s relatively modest success rate may be partially explained by incomplete and biased functional state sampling in the PDB, where predicted motions through evolutionary transfer may correspond to functionally relevant conformational states that have not been structurally resolved and experimental artifacts (for example of crystallographic origin or due to sequence engineering). Our working hypothesis is that a part of the conformational manifold represents functionally relevant motions. To address this challenge, we designed our training loss function specifically to evaluate submanifolds by calculating the minimum error between each reference motion and the set of predicted motions, allowing the model to capture conformational diversity while mitigating the impact of potential artifacts. By comparison, the Atlas MD trajectories represent an easier case, but they are limited to equilibrium distributions of monomeric proteins and do not account for conformational changes induced by partner or ligand binding.

Furthermore, while *PETIMOT*’s predicted motions support competitive conformational ensemble generation through simple isotropic sampling, generative models such as *BioEmu* and *AlphaFlow* offer complementary advantages: trained end-to-end to produce diverse ensembles, they may better capture rare or large-amplitude conformational states, at the cost of significantly higher computational requirements and reduced interpretability.

In addition, our approach is limited to modeling protein motions as linear displacement vectors. While this approximation is sufficient to describe most of the observed conformational heterogeneity, it remains inadequate for modeling highly complex nonlinear deformations. Furthermore, deforming protein structures along a linear motion direction may produce unrealistic conformations at large amplitudes. A possible solution yet to be investigated would be the nonlinear extrapolation techniques widely used in molecular mechanics (Lopéz-Blanco *et al.*, 2011 ▸; Hoffmann & Grudinin, 2017 ▸).

## 6. Conclusion

In this work, we have proposed a new perspective on the problem of capturing protein continuous conformational heterogeneity. Our approach directly infers compact and continuous representations of protein motions. Our comprehensive analysis of *PETIMOT*’s predictive capabilities demonstrates its performance and utility for understanding how proteins deform to perform their functions. It shows that accurate motion subspace prediction, *PETIMOT*’s core strength, provides a strong foundation for modeling protein functional dynamics, while offering interpretability and efficiency advantages over generative models for conformational sampling. *PETIMOT*-generated structures, while not being accurate in a thermodynamic sense, can help practitioners quickly assess possible dynamics or seed other workflows such as heterogeneous cryo-EM reconstruction. Our work opens ways to future developments in protein motion manifold learning, with exciting potential applications in protein engineering and drug development.

## Figures and Tables

**Figure 4:**
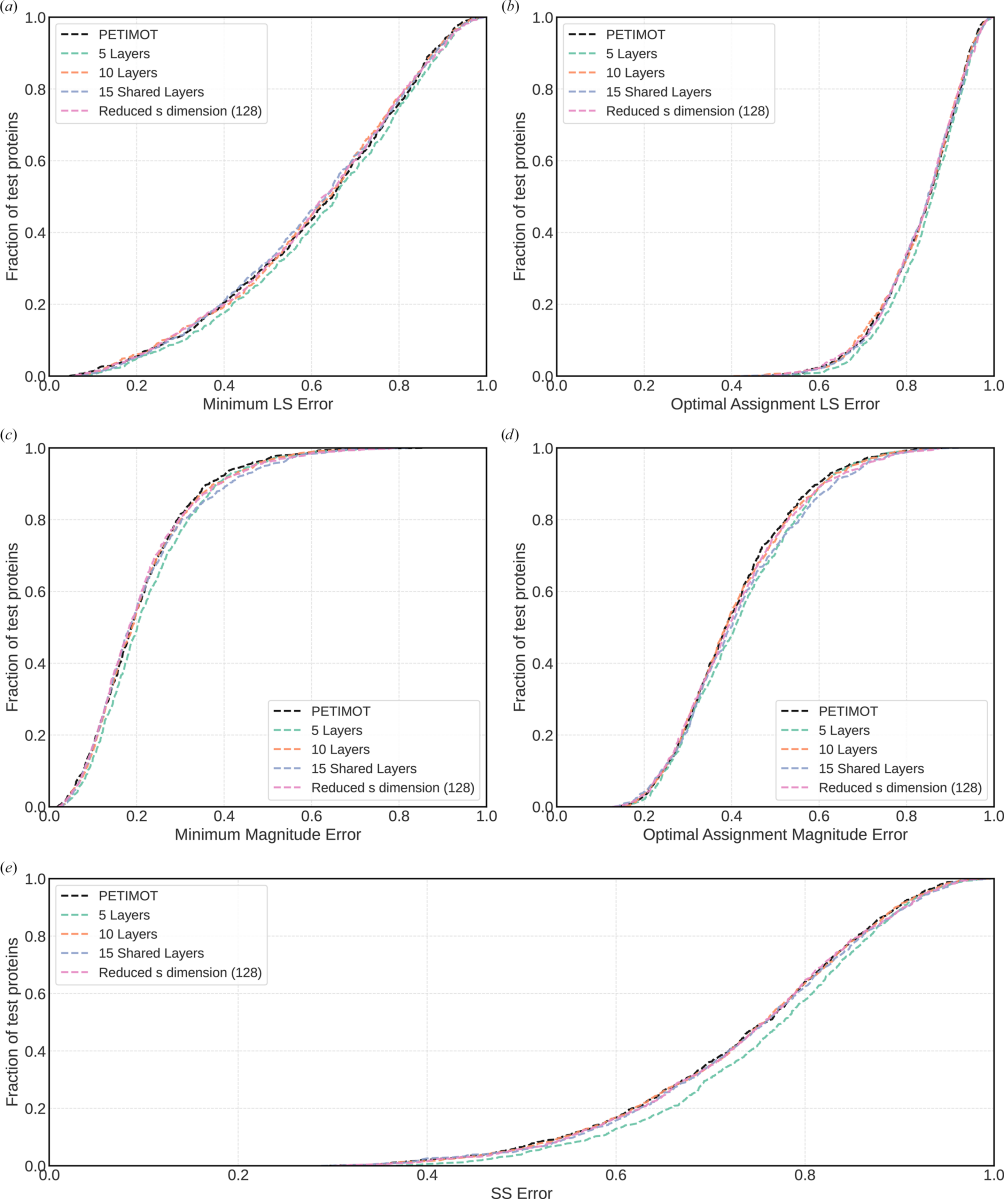
Network depth ablation. We report cumulative curves for LS error (*a*, *b*), magnitude error (*c*, *d*) and SS error (*e*). For each protein, we computed the error either for the best-matching pair of predicted and ground-truth vectors (*a*, *c*) or for the best combination of four pairs of predicted and ground-truth vectors (*b*, *d*). We vary the number of layers in the network and the embedding dimension.

**Figure 11:**
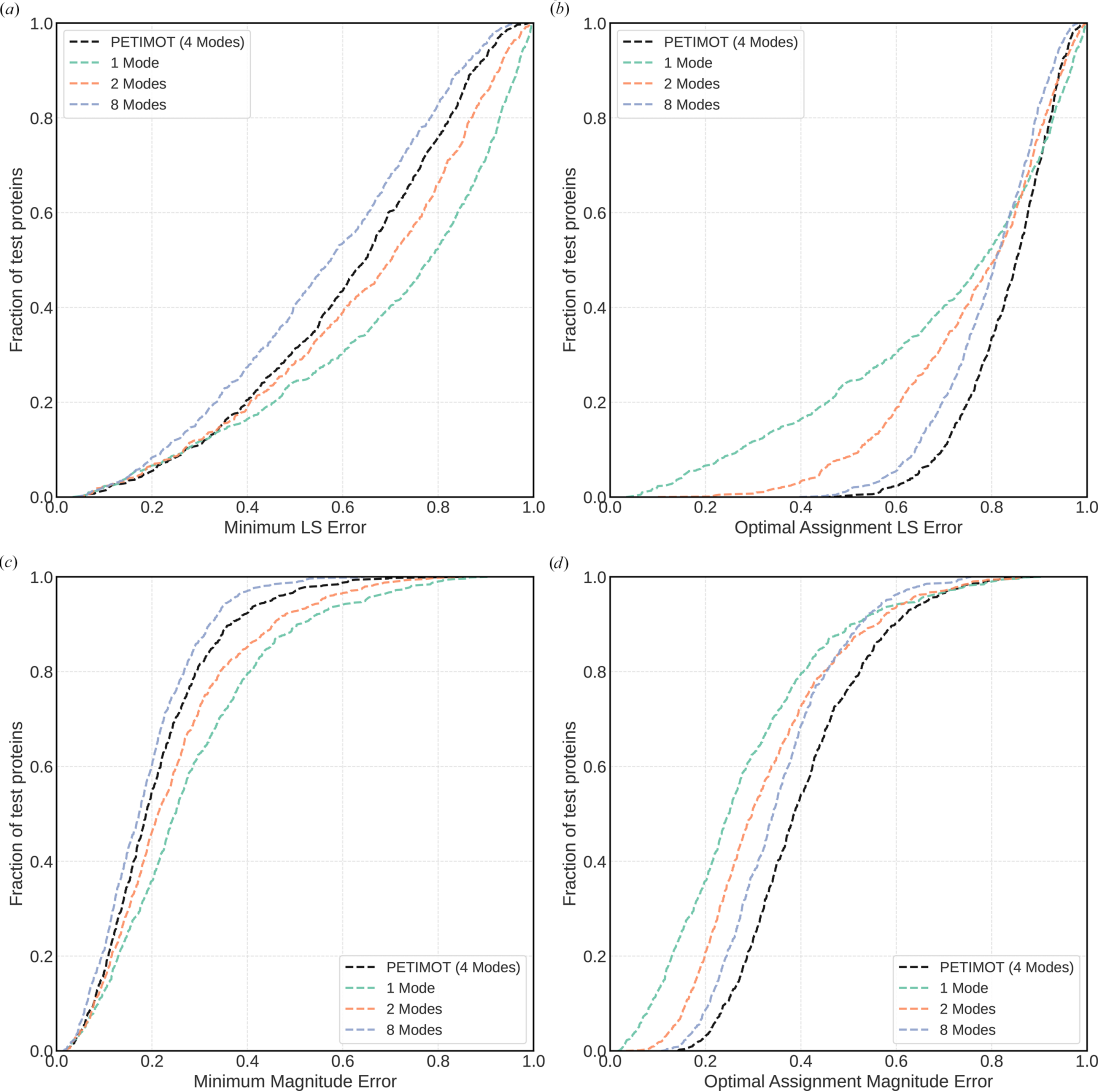
Impact of the number of predicted components. We report cumulative curves for LS error (*a*, *b*) and magnitude error (*c*, *d*). For each protein, we computed the error either for the best-matching pair of predicted and ground-truth vectors (*a*, *c*) or for the best combination of all pairs of predicted and ground-truth vectors using optimal linear assignment (*b*, *d*). We compare models trained to predict different numbers of components (modes): 1, 2, 4 or 8, using only the LS loss.

**Figure 12:**
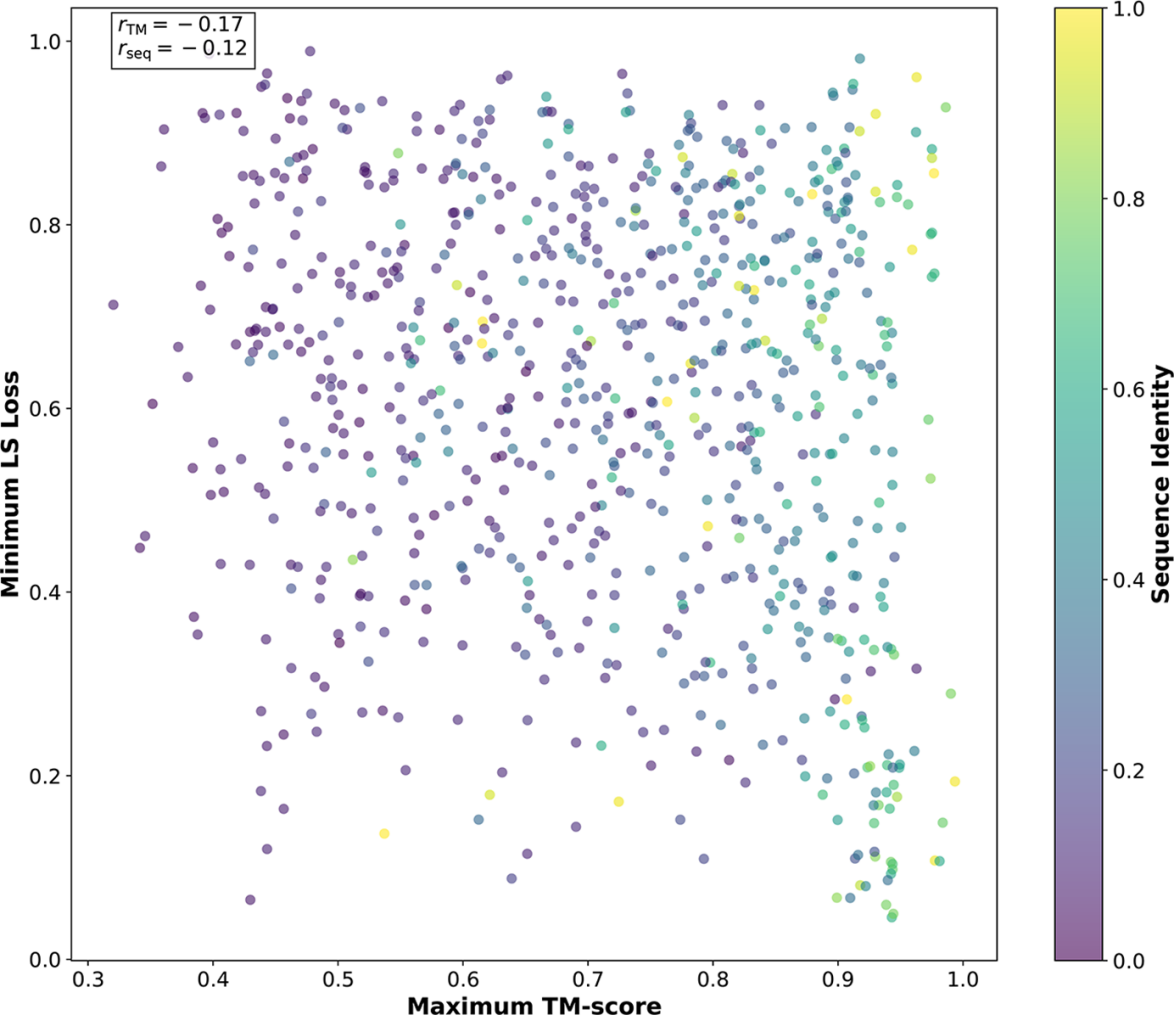
Relationship between *PETIMOT*’s prediction accuracy and structural/sequence similarity with the training set. The minimum LS error is plotted against the maximum TM-score between each test protein and any protein in the training set. Points are colored by the maximum sequence identity to the training samples.

**Figure 13:**
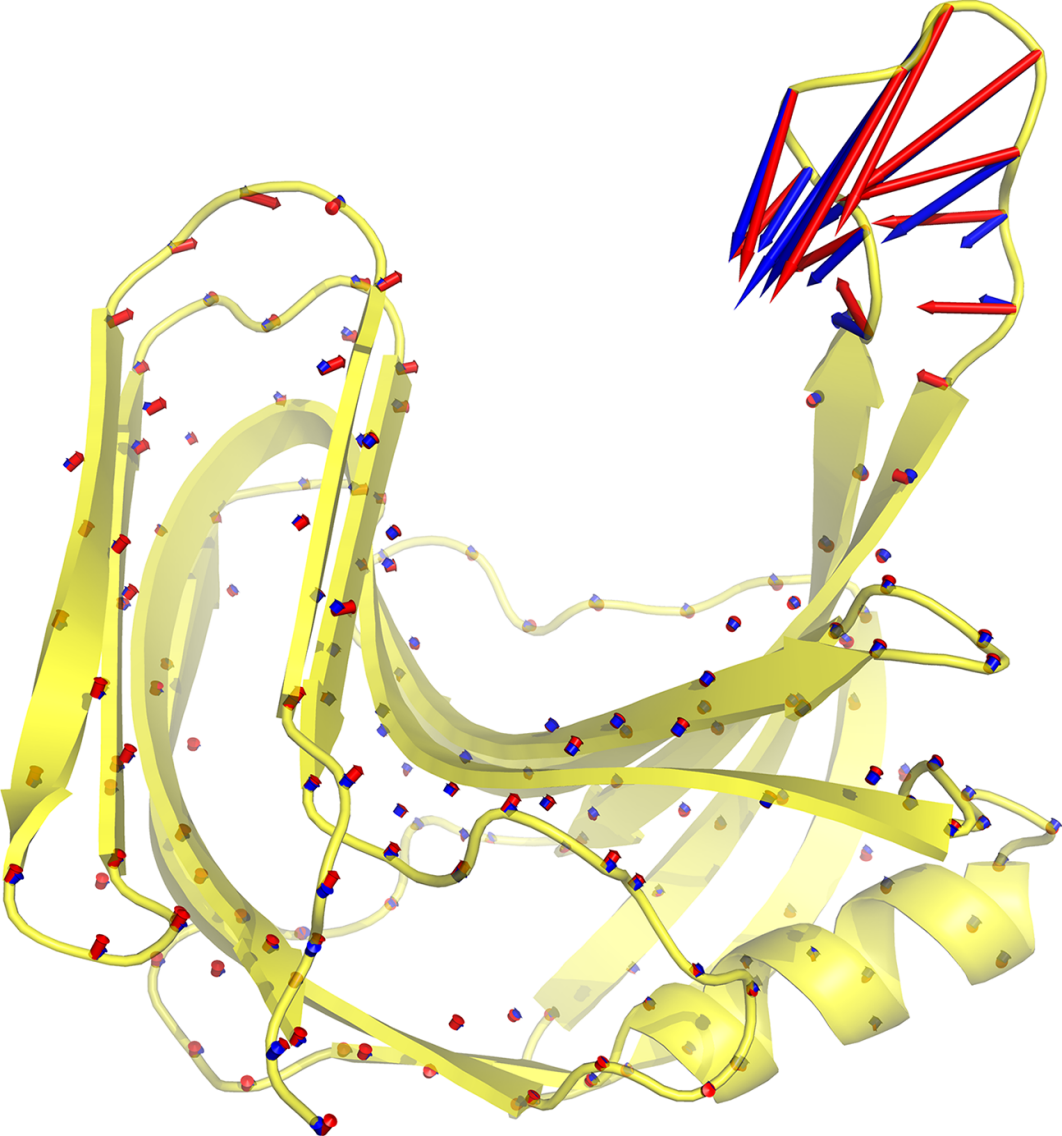
Visualization of predicted (blue arrows) and ground-truth (red arrows) motion vectors for PDB structure 3exu (chain *A*), with an LS error of 0.20. The predicted deformation was used to generate the interpolated conformations shown in Fig. 2 ▸(*b*).

**Figure 14:**
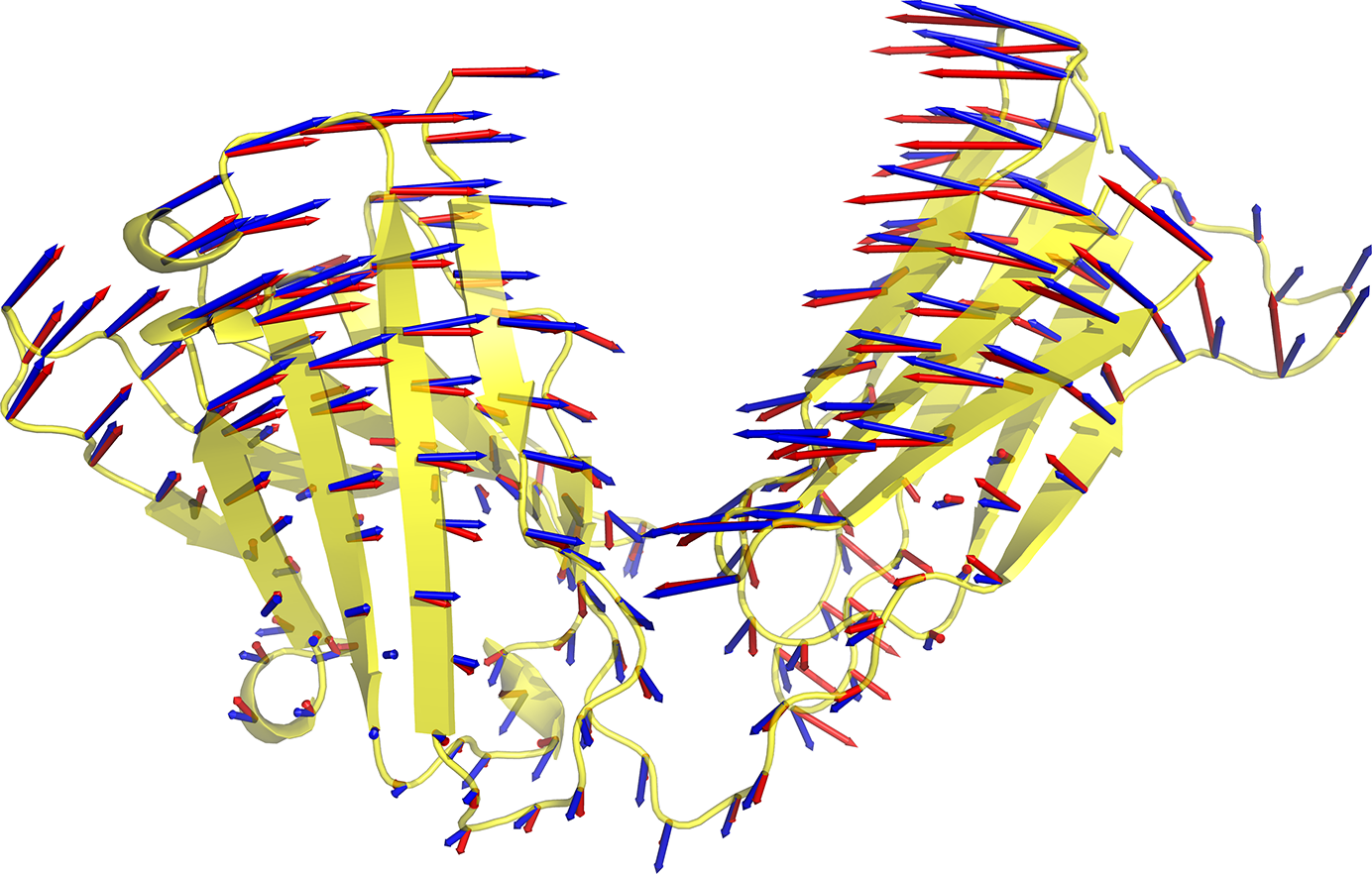
Visualization of predicted (blue arrows) and ground-truth (red arrows) motion vectors for PDB entry 7sd2, with an LS error of 0.18. The predicted deformation was used to generate the interpolated conformations shown in Fig. 2 ▸(*c*).

**Figure 16:**
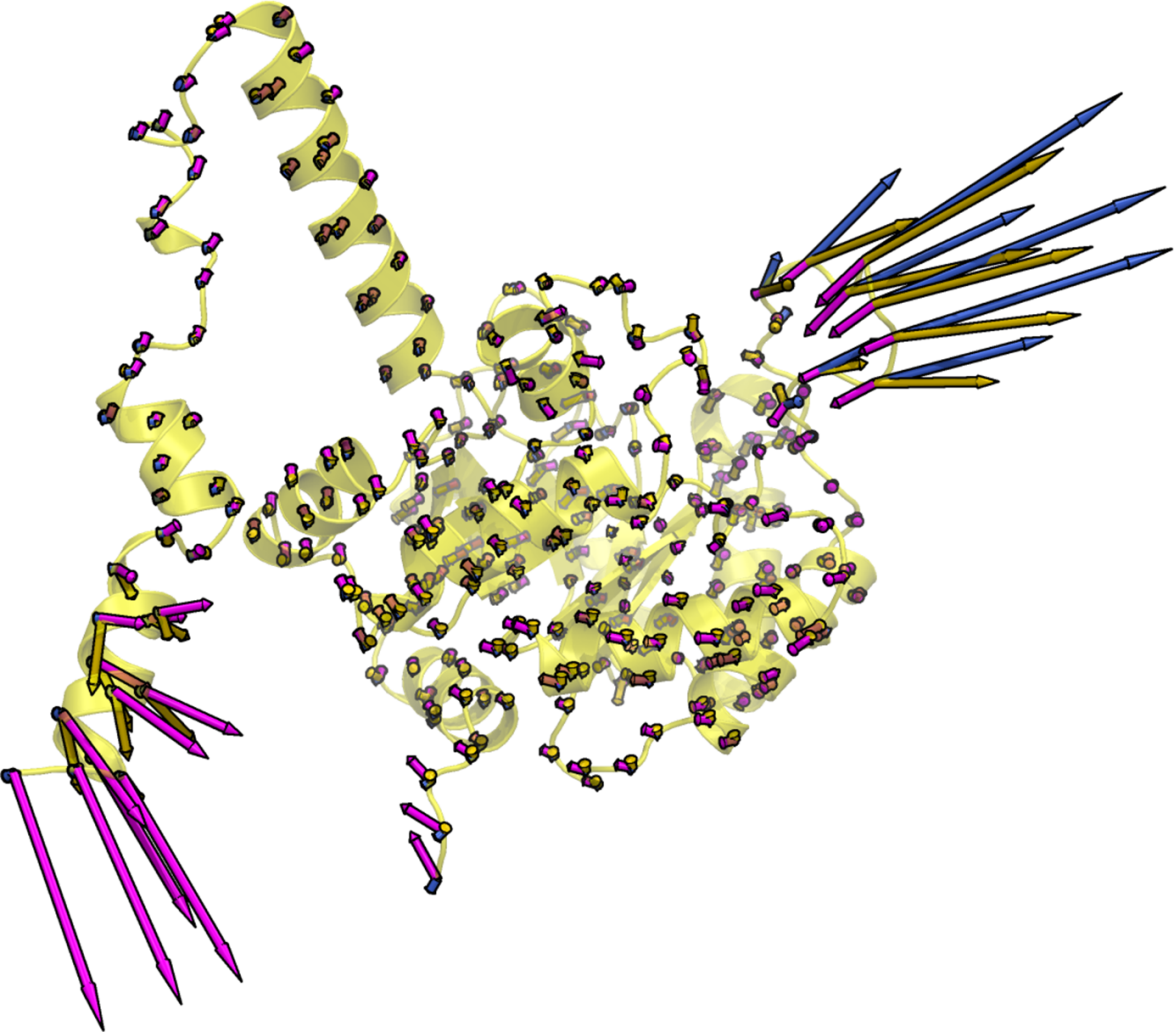
Visualization of ground-truth and predicted motion vectors for PDB entry 1oqf (chain *A*). Ground-truth main motions are depicted as yellow arrows. The best-matching motions predicted by *PETIMOT* (fourth) and *BioEmu* (first) are colored blue and magenta, respectively.

**Table 1:** Comparison of *PETIMOT*’s ability to generate conformational ensembles with respect to generative models *PETIMOT-default* is compared with *AlphaFlow* and *BioEmu* on test824. R.m.s. fluctuation (r.m.s.f.) correlation was computed between conformational samples we generated from the ground-truth or predicted motions. The Min. r.m.s. deviations (r.m.s.d.s) were computed between each of five experimental structures and the predicted ensembles (see Section B5). Arrows indicate whether higher (↑) or lower (↓) values are better; the best results are highlighted in **bold**.

Metrics	*PETIMOT*	*AlphaFlow*	*BioEmu*
R.m.s.f. correlation ↑	**0.59 ± 0.23**	0.51 ± 0.25	0.52 ± 0.27
Coverage, Max./Min. r.m.s.d. <2.5 Å (%) ↑			
Original ensemble†	17.05	25.64	**28.90**
Isotropic sampling	**29.96**	26.12	26.83
Avg Min. r.m.s.d. (Å) ↓			
Original ensemble†	3.52 ± 3.04	4.77 ± 4.99	**4.29 ± 4.34**
Isotropic sampling	**3.46 ± 2.83**	3.54 ± 2.97	3.52 ± 2.95

† For *AlphaFlow* and *BioEmu*, the original ensemble produced by the method; for *PETIMOT*, the input structure with zero displacement.

**Table 2:** Comparison of *PETIMOT-5folds* with the NMA on the full dataset (∼37 000 samples) For each sample, we evaluate the four motions predicted by *PETIMOT* against the four ground-truth motions. We allow the NMA more flexibility by considering either the ten or the four lowest frequency modes. Min. stands for the best-matching pair of predicted and ground-truth vectors. OLA refers to the optimal linear assignment between all predicted and ground-truth vectors. Arrows indicate whether higher (↑) or lower (↓) metrics values are better. The best results are shown in **bold**.

		NMA	
Metrics	*PETIMOT*	First ten modes	First four modes
Success rate (%) ↑	**38.98**	25.43	24.40
Min. LS error ↓	**0.64 ± 0.20**	0.71 ± 0.19	0.72 ± 0.20
Min. magnitude error ↓	**0.23 ± 0.12**	0.24 ± 0.11	0.27 ± 0.14
OLA LS error ↓	**0.84 ± 0.09**	0.85 ± 0.10	0.88 ± 0.09
OLA magnitude error ↓	0.41 ± 0.13	**0.38 ± 0.12**	0.47 ± 0.15
Global SS error ↓	0.75 ± 0.13	**0.67 ± 0.17**	0.79 ± 0.14

**Table 3:** *PETIMOT*’s performance on the test set and comparison with other methods. *PETIMOT-default* is compared with *AlphaFlow*, *BioEmu* and the NMA on test824 For each protein, we evaluate the four motions predicted by *PETIMOT* or inferred from *AlphaFlow*/*BioEmu*-predicted ensembles against the four ground-truth motions. We allow the NMA more flexibility by considering the ten lowest frequency modes. Minimum indicates the best-matching pair of predicted and ground-truth motions. OLA refers to the optimal linear assignment between all predicted and ground-truth vectors. Arrows indicate whether higher (↑) or lower (↓) values are better; the best results are highlighted in **bold**. The running times were measured on an Intel Xeon W-2245 CPU @ 3.90 GHz equipped with a GeForce RTX 3090 for *PETIMOT*, *AlphaFlow* and the NMA, and on an AMD Ryzen 9 7950X 16-Core CPU @ 5.88 GHz equipped with an NVIDIA RTX A6000 for *BioEmu*, which can be up to 30% faster in some tasks.

				NMA	
Metrics	*PETIMOT*	*AlphaFlow*	*BioEmu*	First ten modes	First four modes
Running time ↓	**15.82 s**	38 h 7 min	39 h 12 min	43.59 s	
Success rate (%) ↑	**43.57**	31.80	31.34	25.73	24.88
Minimum LS error ↓	**0.61 ± 0.22**	0.68 ± 0.21	0.68 ± 0.20	0.70 ± 0.19	0.72 ± 0.20
Minimum magnitude error ↓	**0.21 ± 0.12**	0.24 ± 0.12	0.23 ± 0.12	0.25 ± 0.11	0.27 ± 0.14
OLA LS error ↓	**0.83 ± 0.10**	0.86 ± 0.10	0.86 ± 0.10	0.85 ± 0.10	0.88 ± 0.10
OLA magnitude error ↓	0.41 ± 0.14	0.43 ± 0.14	0.42 ± 0.13	**0.39 ± 0.12**	0.48 ± 0.15
Global SS error ↓	0.73 ± 0.14	0.78 ± 0.14	0.77 ± 0.14	**0.67 ± 0.16**	0.79 ± 0.14

**Table 4:** Comparison between *PETIMOT* and NMA variants *PETIMOT-default* is compared with NMA variants on test824. For each sample, we evaluate the four motions predicted by *PETIMOT* against the four ground-truth motions. We allow the NMA more flexibility by considering the ten lowest frequency modes. We vary the distance cutoff used to defined the elastic network model and its resolution (C^α^ atoms only or all atoms) across NMA variants. Minimum indicates the best-matching pair of predicted and ground-truth vectors. OLA refers to the optimal linear assignment between all predicted and ground-truth vectors. Arrows indicate whether higher (↑) or lower (↓) metric values are better. The best results are shown in **bold**.

Method	*PETIMOT*	NMA			
Resolution	C^α^ only	C^α^ only			All-atom
Cutoff distance		7.5 Å	10 Å	13 Å	5 Å
Success rate (%) ↑	**43.57**	19.05	25.73	24.27	28.52
Minimum LS error ↓	**0.61 ± 0.22**	0.75 ± 0.19	0.70 ± 0.19	0.71 ± 0.19	0.69 ± 0.19
Minimum magnitude error ↓	**0.21 ± 0.12**	0.28 ± 0.12	0.25 ± 0.11	0.25 ± 0.12	0.23 ± 0.11
OLA LS error ↓	**0.83 ± 0.10**	0.88 ± 0.09	0.85 ± 0.10	0.85 ± 0.10	0.84 ± 0.10
OLA magnitude error ↓	0.41 ± 0.14	0.42 ± 0.13	0.39 ± 0.12	0.40 ± 0.13	**0.37 ± 0.12**
Global SS error ↓	0.73 ± 0.14	0.73 ± 0.16	0.67 ± 0.16	0.68 ± 0.17	**0.66 ± 0.16**

## Data Availability

The code and the data are available at https://github.com/PhyloSofS-Team/PETIMOT.

## Appendix A Invariance of the proposed losses

Theorem A.1 SS loss is invariant under unitary transformations of *X* and *Y* subspaces.

Proof Without loss of generality, let us assume that we apply a unitary transformation  to a subspace , such that the result *X*′ = *X*^⊥^*U*, with , spans the same subspace as *X*^⊥^, as it is a linear combination of the original basis vectors from *X*^⊥^. Then, let us rewrite the SS loss as

As the Frobenius matrix norm is invariant under orthogonal, or more generally, unitary, transformations,  = , which completes the proof. □

Corollary A.1.1 The SS loss is invariant to the direction permutations in the Gram–Schmidt orthogonalization process.

Proof Let us consider two linear subspaces  and  resulting from the Gram–Schmidt orthogonalization of *X*, where we arbitrarily choose the order of the orthogonalization vectors. Both  and  will span the same subspace as *X*, and since both  and  are also orthogonal, one is a unitary transformation of the other, , which completes the proof. □

Theorem A.2 IS loss is invariant under unitary transformations of *X* and *Y* subspaces.

Proof Following the previous proof, without loss of generality, let us assume that we apply an orthogonal (unitary) transformation  to a subspace , such that the result *X*′ = *XU*, with , spans the same subspace as *X*. Then, let us rewrite the IS loss as

As the Frobenius matrix norm is invariant under orthogonal transformations,  and , which completes the proof. □

## Appendix B Methods details

### b1. Training data

#### b1.1. Conformational collections

To generate the training data, we used *DANCE* (Lombard *et al.*, 2024 ▸) to construct a nonredundant set of conformational collections representing the entire PDB as of June 2023. Wherever possible, we enhanced the data quality by replacing raw PDB coordinates with their updated and optimized counterparts from *PDB-REDO* (Joosten *et al.*, 2014 ▸). Each conformational collection was designed to include only closely related homologs, ensuring that any two protein chains within the same collection shared at least 80% sequence identity and coverage. Collections with insufficient data points were excluded as we require at least five conformations. To simplify the data, we retained only C^α^ atoms (option -c) and accounted for coordinate uncertainty by applying weights (option -w). To reduce structural redundancy of crystallo­graphic artifacts and nonbiological conformations, we removed any conformation *A* deviating by less than 0.1 Å from another one *B*, provided that the sequence of *A* is identical to or included in that of *B*.

#### b1.2. Handling missing data

The conformations in a collection may have different lengths, reflected by the introduction of gaps when aligning the amino-acid sequences. As detailed in Lombard *et al.* (2024 ▸), we fill these gaps with the coordinates of the conformation used to center the data. The imputed positions have zero-centered coordinates and therefore contribute no variance to the decomposition, making this a fairly neutral imputation strategy. Moreover, to explicitly account for data uncertainty, we assign confidence scores to the residues and include them in the structural alignment step and the eigendecomposition. The confidence score of a position *i* reflects its coverage in the alignment,

where “X” is the symbol used for gaps and *m* is the number of conformations. The structural alignment of the *j*th conformation onto the reference conformation amounts to determining the optimal rotation that minimizes the following function (Kabsch, 1976 ▸; Kearsley, 1989 ▸),

where  is the *i*th centered coordinate of the *j*th conformation and  is the *i*th centered coordinate of the reference conformation. The resulting aligned coordinates are then multiplied by the confidence scores prior to the PCA, as we explain below.

The weighting scheme effectively prevents large deviations in uncertain regions, typically highly localized loop motions, from dominating the variance (Lombard *et al.*, 2024 ▸).

#### b1.3. Eigenspaces of positional covariance matrices

The Cartesian coordinates of each conformational ensemble can be stored in a matrix *R* of dimensions 3*N* × *m*, where *N* is the number of residues (or positions in the associated multiple sequence alignment) and *m* is the number of conformations. Each position is represented by a C^α^ atom. We compute the coverage-weighted (to account for missing data, as explained above) covariance matrix as in equation (2). The covariance matrix is a 3*N* × 3*N* square matrix, symmetric and real.

We decompose *C* as *C* = *VDV*^T^, where *V* is a 3*N* × 3*N* matrix with each column defining a sqrt-coverage-weighted eigenvector or a principal component that we interpret as a *linear motion*. *D* is a diagonal matrix containing the eigen­values. Specifically, the *k*th principal component was expressed as a set of 3D (sqrt-coverage-weighted) displacement vectors  for the *L* C^α^ atoms of the protein residues. To enable cross-protein comparisons, the vectors were normalized such that . The sum of the eigenvalues  amounts to the total positional variance of the ensemble (measured in Å^2^) and each eigenvalue reflects the amount of variance explained by the associated eigenvector.

#### b1.4. Data augmentation

The reference conformation used to align and center the 3D coordinates corresponds to the protein chain with the most representative amino-acid sequence. To increase data diversity, four additional reference conformations were defined for each collection. At each iteration, the new reference conformation was selected as the one with the highest r.m.s.d. relative to the previous reference. This iterative strategy maximizes the variability of the extracted motions by emphasizing the impact of changing the reference.

### b2. Message passing

The node embeddings and predicted motion vectors are updated iteratively according to the following Algorithm B1, where MessageMLP stands for one hidden layer of size 256, followed by a GELU activation and a dropout layer with rate 0.4.

### b3. SE(3)-equivariant features

We represent protein structures as attributed graphs. The node embeddings are computed with the pre-trained protein language model *ProstT*5 (Heinzinger *et al.*, 2024 ▸). It is a fine-tuned version of the sequence-only model *T*5 that translates amino-acid sequences into sequences of discrete structural states and reciprocally.

The edge embeddings are computed using SE(3)-invariant features derived from the input backbone, similarly to prior work (Ingraham *et al.*, 2019 ▸, 2023 ▸; Dauparas *et al.*, 2022 ▸). Specifically, the features associated with the edge *e*_*ij*_ from node (atom) *i* to node (atom) *j* are the following.

(i) *Quaternion representation*. A four-dimensional quaternion encoding the relative rotation *R*_*ij*_ between the local reference frames of residues *i* and *j*.
(ii) *Relative translation*. A three-dimensional vector representing the translation  between the local reference frames.
(iii) *Chain separation*. The sequence separation between residues *i* and *j*, encoded as log(|*i* − *j*| + 1).
(iv) *Spatial separation*. The logarithm of the Euclidean distance between residues *i* and *j*, computed as , where ε = 10^−8^.
(v) *Backbone atom distances*. Distances between all backbone atoms (N, C^α^, C, O) at residues *i* and *j*, encoded through a radial basis expansion. For each pairwise distance *d*_*ab*_, we compute
  where  are centers spaced linearly in [0, 20] Å and σ = 1 Å. This creates a 16 × 20 = 320-dimensional feature vector, as we have 16 pairwise distances (4 × 4 atoms) each expanded in 20 basis functions.

### b4. Training procedure

For the *default* version, we randomly split the 7335 conformational collections defined with *DANCE* into training, validation and test sets with a 70:15:15 ratio. The data-augmentation procedure resulted in 5119 × 5 = 25 595 training samples and 1099 × 5 = 5495 validation samples.

For the *stringent* version, we considered clusters of protein chains defined at 30% sequence identity and 80% sequence coverage using *MMseqs*2 (Steinegger & Söding, 2017 ▸). These clusters define distant protein families and we refer to them as clus-30 in the following. We kept the test proteins used *PETIMOT-default* as is and we removed all collections belonging to the same clus-30 clusters as these proteins from the training and validation sets. Then, we re-defined a training–validation random split at the level of the clus-30 clusters with a 9:1 ratio. This operation ensures that any pair of training–validation, training–test or validation–test collections do not share more than 30% sequence identity. Finally, for each training or validation clus-30 cluster, we randomly drew five samples. This step ensures that each protein family is evenly represented in the training and validation sets. We also redundancy-reduced test824, keeping only one protein for each clus-30 cluster, which led to 734 proteins (test734).

For the *5fold* version, we conducted a fivefold cross-validation experiment with strict similarity filtering over the full training set comprising 36 675 samples. We ensured a two-stage similarity filtering process.

(i) *Structural similarity removal*. First, we used *FoldSeek* (Van Kempen *et al.*, 2024 ▸) to cluster protein chains using an *e*-value threshold of 1 × 10^−2^. Any two chains belonging to two different clusters do not share significant structural similarity.
(ii) *Sequential similarity removal*. We randomly partitioned the dataset into five folds and applied a cross-validation procedure. We implemented an additional sequence similarity-based filtering using *MMseqs*2. Specifically, for each fold, we removed from the training set (80% of the data) the protein chains sharing more than 30% sequence identity with any of the chains from the test set (20%).

The models were optimized using *AdamW* (Loshchilov & Hutter, 2019 ▸) with a learning rate of 5 × 10^−4^ and a weight decay of 0.01. We employed gradient clipping with a maximum norm of 10.0 and mixed precision training with PyTorch’s Automatic Mixed Precision. The learning rate was adjusted using the PyTorch ReduceLROnPlateau scheduler, which monitored the validation loss, reducing the learning rate by a factor of 0.2 after ten epochs without improvement. Training was performed with a batch size of 32 for both training and validation sets. We implemented early stopping with a patience of 50 epochs, monitoring the validation loss. The model achieving the best validation performance was selected for final evaluation.

We trained the model on a single NVIDIA A100-SXM4-80GB GPU. One epoch took about 9 min of real time.

### b5. Evaluation and comparison with other methods

We primarily evaluated *PETIMOT* on a test set of 1117 proteins, reduced to 824 (test824) to comply with the requirements of other methods (see below). In addition, our fivefold cross-validation training procedure allowed us to evaluate the predictive capacity of *PETIMOT* on the full dataset of 36 675 samples and systematically compare it with the NMA (Table 2 ▸).

#### b5.1. Evaluation protocol and metrics

We primarily relied on three metrics defined in the main text to evaluate *PETIMOT* and compare it with other methods: the LS error, the magnitude error and the global SS error. We computed the LS error and the magnitude error either on the best-matching pair of predicted and ground-truth motions (Min.) or on the full set of matched pairs determined through optimal linear assignment (OLA). In addition, we included several commonly used metrics for assessing conformational ensembles: r.m.s.f. correlation and minimum r.m.s.d. to experimental structures.

To generate conformational ensembles, we deformed each test protein’s 3D coordinates along *PETIMOT*’s four predicted motions. Since we do not have access to the oracle eigenvalues at inference, we sampled displacement coefficients uniformly on a hypersphere of fixed radius. Formally, given *K* predicted modes assembled in the matrix , a conformational sample is generated as

where  are the C^α^ coordinates of the input reference structure and *E*_total_ = ε · *N* is a total energy budget scaling linearly with the number of residues *N*. This formulation ensures that all modes contribute equally in expectation, without imposing any ordering or relative weighting among predicted modes. We set ε = 1 in all experiments, giving a displacement scale of (*N*)^1/2^ Å. This choice is motivated by the substantial range of conformational diversity observed in the experimental collections of our benchmark, with a mean r.m.s.d. of 3.70 ± 3.36 Å between conformations within each ensemble and a median maximum r.m.s.d. of 3.73 Å across ensembles (up to 40.21 Å).

For r.m.s.f. correlation, we additionally generated ensembles from ground-truth motions and corresponding eigen­values. We randomly sampled deformation amplitudes from a Gaussian distribution with variance proportional to each ground-truth motion’s eigenvalue. We then compared per-residue fluctuations between predicted and ground-truth ensembles. For experimental structure coverage, we leveraged our iterative strategy for data augmentation (Section *B*1) to select five diverse conformations from the ground-truth test collections. Then, for each experimental structure, we computed its minimum r.m.s.d. to the closest conformation in the predicted motion-derived ensemble. We defined coverage as the fraction of test proteins for which all five structures have a miminum r.m.s.d. below 2.5 Å, a threshold commonly used for assessing conformational similarity in C^α^-only comparisons.

#### b5.2. Comparison with the normal-mode analysis

We compared our approach with the physics-based un­supervised normal-mode analysis (NMA) method (Hayward & Go, 1995 ▸). The NMA takes as input a protein 3D structure and builds an elastic network model where the nodes represent the atoms and the edges represent springs linking atoms located close to each other in 3D space. The four lowest normal modes are obtained by diagonalizing the mass-weighted Hessian matrix of the potential energy of this network. We used the highly efficient *NOLB* method, version 1.9, downloaded from https://team.inria.fr/nano-d/software/nolb-normal-modes/ (Hoffmann & Grudinin, 2017 ▸), to extract the first *K* normal modes from the test protein 3D conformations. Specifically, we used the following command by default, where the elastic network is defined using a distance cutoff (option -c) of 10 Å and the input PDB file contains only C^α^ atoms. Additionally, we tested the impact of varying the cutoffs (7.5 Å, 13 Å) and of considering all atoms, using *SCWRL*4 (Krivov *et al.*, 2009 ▸) to reconstruct the side chains trimmed during data pre-processing. We evaluated the NMA using exactly the same metrics and protocols as those used for *PETIMOT*, since both methods output a set of linear motions. While we retained only the first four NMA modes for our primary evaluation, we additionally explored the impact of including all ten first modes when computing the evaluation metrics.

#### b5.3. Comparison with generative models

We considered the flow-matching based framework *AlphaFlow* and the diffusion-based *Biomolecular Emulator* (*BioEmu*) as additional baselines.

##### b5.3.1. Conformational ensemble generation with *AlphaFlow*

Out of a total of 1117 proteins comprised in our test set, we excluded 293 proteins because they were too long (>450 amino acids) to be handled by *AlphaFlow* in a reasonable amount of time using our computing resources. We downloaded the distilled ‘PDB’ models from https://github.com/bjing2016/alphaflow. We executed *AlphaFlow* using the following command:*AlphaFlow* relies on *OpenFold* (Ahdritz *et al.*, 2024 ▸) to retrieve the input multiple sequence alignment (MSA).

We used *AlphaFlow* to generate 50 conformations for each test protein.

##### b5.3.2. Conformational ensemble generation with *BioEmu*

We ran *BioEmu* through its Python API by reading the input FASTA file then launching the *sample* function as *BioEmu* generated up to 50 conformations per test protein, with an average of 44 ± 8 conformations.

##### b5.3.3. Motion and conformation comparisons

While *PETIMOT* predicts a set of linear motions from an input protein sequence and structure, *AlphaFlow* and *BioEmu* predict a conformational ensemble from an input protein sequence. As a consequence, the input structure given to *PETIMOT* might not lie within the conformational ensemble that predicted the generative models and our evaluation framework needs adaptation to ensure fair comparison. Firstly, to directly compare motions with our three main metrics, we aligned all members of the *AlphaFlow* or *BioEmu* original ensembles with the test protein conformations, with identity coverage weights, and extracted the principal linear motions. Secondly, to compare conformational ensembles using r.m.s.f. and r.m.s.d. metrics, we considered the original ensembles outputted by *AlphaFlow* or *BioEmu*, and, in addition, we generated new ensembles by deforming each test protein along the previously extracted principal linear motions. For this, we used the same ‘isotropic’ sampling protocol as that used for *PETIMOT* conformational ensemble generation.

We additionally mention that we did not filter or adapt our test set to the generative models. As a consequence, there can be data leakage between *AlphaFlow* and *BioEmu* training data and our test examples.

#### b5.4. Running times

The running times were measured on an Intel Xeon W-2245 CPU @ 3.90 GHz equipped with GeForce RTX 3090 for *PETIMOT*, *AlphaFlow* and the NMA, and on an AMD Ryzen 9 7950X 16-Core CPU @ 5.88 GHz equipped with NVIDIA RTX A6000 for *BioEmu*. We note that in deep architectures RTX A6000 can be up to 1.3 times faster compared with RTX 3090.

### b6. Ablation studies

To understand the impact of different components on the performance of our model, we carried out ablation studies. We list them below.

#### b6.1. Model architecture variations

(i) *Network depth*. We experimented with different numbers of message-passing layers (five and ten layers compared with our default value of 15 layers).
(ii) *Layer sharing*. We tested a variant where all message-passing layers share the same parameters, as opposed to our default where each layer has unique parameters.
(iii) *Reduced internal embedding dimension*. We tested a model with a smaller internal embedding dimension of 128 instead of the default 256.

Fig. 4 ▸ shows the evaluation of these modifications. A shallow five-layers network underperforms on all evaluation metrics. The difference between other variants is not very significant.

#### b6.2. Structure and sequence information ablation

(i) *Structure ablation*. We removed all structural information from the model to assess the importance of geometric features and the performance with the protein language model embeddings only. We performed this by removing the edge attributes of the input of the message-passing MLP.
(ii) *Sequence ablation*. We ablated sequence information by replacing protein language model embeddings with random embeddings, testing them both with and without structural information.
(iii) *Embedding variants*. We evaluated a different protein language model (*ESM-Cambrian* 600M), both with and without structural tokens.

The evaluation results are shown in Fig. 5 ▸. The results demonstrate that while both *ProstT*5 and *ESM-Cambrian* 600M perform similarly when combined with structural information, removing structural features leads to markedly different outcomes. *ProstT*5 embeddings partially compensate for the missing structural information, likely due to their structure-aware training, while relying solely on *ESM-C* embeddings results in poor performance.

#### b6.3. Problem formulation ablation

We analyzed different combinations of our loss terms (compared with our default balanced weights of LS + SS).

(i) *Least-square loss (LS)*. Using only the LS loss (weight 1.0).
(ii) *Squared sinus loss (SS)*. Using only the SS loss (weight 1.0).
(iii) *Independent subspaces (IS)*. Using only the IS loss (weight 1.0).

Figs. 6 ▸ and 7 ▸ compare three individual losses with the default option. The IS problem formulation underperforms on all the metrics but the global SS error. The default LS + SS formulation performs slightly better than those with individual loss components.

#### b6.4. Graph connectivity ablation

We investigated different approaches to constructing the protein graph.

(i) *Nearest neighbor only*. Using 15 nearest neighbors (sorted according to the corresponding C^α^–C^α^ distances) without random edges.
(ii) *Random connections only*. Using 15 random edges without nearest neighbors. This set is updated between every layer at each epoch.
(iii) *Static connectivity*. Using a fixed set of random neighbors between the layers. This set is updated at each epoch.

Fig. 8 ▸ shows the ablation results. We can see that the nearest neighbor-only setup underperforms on all the metrics. Among other options, the random connectivity-only option obtains lower results at higher metric values. The default option performs on a par with the static connectivity, showing slightly better results on the optimal assignment magnitude error metrics.

### b7. Additional validation on ATLAS molecular-dynamics data

To further assess the generalization capabilities of our model, we conducted an additional validation experiment using molecular-dynamics (MD) data from the ATLAS database. This experiment provided an independent test of *PETIMOT*’s performance on high-quality data with distinct characteristics from our training data.

The *ATLAS* MD dataset underwent a systematic preprocessing pipeline. Firstly, we extracted the principal components from the MD trajectories. Subsequently, we assigned samples to appropriate cross-validation folds, ensuring strict exclusion of both structural and sequential similarity between training and test sets. This rigorous assignment process yielded 400 samples suitable for evaluation. The inference was then performed by applying our trained *PETIMOT* models to predict the conformational motions for each sample. Fig. 9 ▸ shows the association between minimum LS error and SS error.

## Appendix C Additional results

### c1. Generalization and robustness

The performances achieved by *PETIMOT-default* on test824 (Table 3 ▸, success rate of 43.57%) generalize to the full dataset with *PETIMOT-5folds* (Table 2 ▸, success rate of 38.98%). Moreover, *PETIMOT* consistently outperforms the NMA on both test824 and the full dataset.

We further assessed the robustness of *PETIMOT*’s performance across different reference conformations derived from the same experimental collection. When *PETIMOT-5folds* predicted at least one acceptable motion, with minimum LS below 0.6, for a given collection, it did so for at least two out of five reference conformations in almost 80% of cases (Fig. 10 ▸*a*). It predicted acceptable motions for all five reference conformations in 28% of cases. These results suggest limited sensitivity of *PETIMOT* performance to the choice of reference conformation. Furthermore, *PETIMOT-5folds* is more robust to the choice of reference conformation than the NMA (Fig. 10 ▸*b*). When both *PETIMOT-5folds* and the NMA predicted at least one acceptable motion for a given collection, *PETIMOT-5folds* did so for more conformations than the NMA in about half of the cases. The NMA predicted acceptable motions for more conformations in only 17% of the cases.

### c2. Comparison with other methods

Table 3 ▸ provide a comprehensive comparison of *PETIMOT* with *AlphaFlow*, *BioEmu* and NMA variants on motion-related metrics. *PETIMOT* achieves the highest success rate (43.57%) and consistently outperforms all baselines on minimum and OLA error metrics. The NMA with ten modes remains competitive on OLA magnitude error and global SS error, metrics that aggregate over all predicted motions and may therefore favor methods with access to a larger mode set, while *PETIMOT* leads on all metrics directly reflecting the quality of individual motion predictions. Across all methods, *PETIMOT* is also by far the fastest, running in under 16 s per protein compared with over 38 h for *AlphaFlow* and *BioEmu*.

### c3. Comparison with NMA variants

We compared *PETIMOT* with several NMA variants (Table 4 ▸). We explored several distance cutoffs to define the elastic network model (ENM). The cutoff of 10 Å yields the best performance (success rate 25.73%, minimum LS error 0.70), while smaller (7.5 Å) and larger (13 Å) cutoffs perform slightly worse, suggesting a moderate sensitivity to this hyperparameter. In addition, we increased the resolution of the ENM by including all atoms. For evaluation, all-atom NMA displacement vectors are assessed at C^α^ positions only, ensuring that metrics remain directly comparable across all methods. This variant achieves the best NMA performance overall on several metrics, including success rate (28.52%) and OLA magnitude error (0.37), as well as global SS error (0.66). However, the differences with respect to the best performing C^α^-based NMA (at 10 Å cutoff) remain small and hence increasing the ENM resolution does not affect the conclusions of our evaluation. *PETIMOT* outperforms all NMA variants on the three primary metrics, success rate (43.57%), minimum LS error (0.61) and minimum magnitude error (0.21), with consistent margins, while the NMA variants perform competitively on OLA magnitude error and global SS metrics. This is consistent with the asymmetry in the comparison setup: NMA is allowed ten modes while *PETIMOT* predicts only four, which may favor NMA on metrics that aggregate over all predicted motions.

### c4. Influence of the number of predicted components

We also experimented with a different number of predicted components, while maintaining the number of ground-truth components fixed at *L* = 4. For these experiments, we trained additional models with the LS loss only.

(i) Single component prediction (one mode).
(ii) Reduced component prediction (two modes).
(iii) Extended component prediction (eight modes).

We compare these with our default setting of *K* = 4 predicted components. Fig. 11 ▸ shows the results. The key insight is that minimum-based metrics (Figs. 11 ▸*a* and 11 ▸*c*) and assignment-based metrics (Figs. 11 ▸*b* and 11 ▸*d*) measure different aspects of subspace quality. Minimum metrics measure the best possible match between any predicted and ground-truth component. These improve with more predicted components (from one to eight) because having more candidates increases the likelihood of finding at least one good match with each ground-truth component. Optimal assignment metrics measure overall subspace alignment by finding the best one-to-one matching between predicted and ground-truth components. Here, models with fewer predicted components (1–2) perform better because they face fewer constraints in the assignment problem: each predicted component can be matched to the best available ground-truth component without competition. The eight-component model maintains the best performance overall, as having more candidate vectors provides flexibility while still capturing the four-dimensional ground-truth subspace effectively.

Fig. 12 ▸ compares the accuracy of the predicted test proteins (minimum LS loss) with the structural (TM-score) and sequence (sequence identity) distances to the training set. We do not see a clear correlation between the prediction accuracy and the similarity to the training examples. Please also see Figs. 2 ▸(*b*) and 2 ▸(*c*) for comparison.

### c5. Visualization of the predictions

Figs. 13 ▸ and 14 ▸ show predicted (blue arrows) and ground-truth (red arrows) motion vectors for the xylanase A from *Bacillus subtilis* and the periplasmic domain of gliding motility protein GldM from *Capnocytophaga canimorsus*, respectively. Fig. 15 ▸ shows predicted motion vectors.

Fig. 16 ▸ show the ground-truth main motion (yellow arrows) exhibited by experimental structures of the enzyme 2-methylisocitrate lyase (PrpB) from *Escherichia coli* and the best-matching predictions from *PETIMOT* (blue) and *BioEmu* (magenta). This enzyme, which catalyses the last step of the methylcitrate cycle, belongs to the isocitrate lyase protein family. Members of this family share an opening–closing loop mechanism associated with ligand binding. *PETIMOT* captures this loop motion very precisely (minimum LS error below 0.4), while the *BioEmu* ensemble mostly exhibits motions of the C-terminal helix.

## Appendix D Licenses for used resources

In this work, we use several existing resources. The protein structures were obtained from the Protein Data Bank (PDB; https://www.rcsb.org/, version accessed on June 2023), which is distributed under the CC0 1.0 Universal Public Domain Dedication License (CC0 1.0). We complemented PDB data with data from *PDB-REDO* (accessed June 2023) developed by Joosten *et al.* (2014 ▸), available at https://pdb-redo.eu under the license specified at https://pdb-redo.eu/license. For protein language modeling, we employed *ProstT*5 developed by Heinzinger *et al.* (2024 ▸), available under the MIT license at https://huggingface.co/Rostlab/ProstT5, and *ESM-Cambrian* 600M (version esmc-600m-2024-12) developed by the EvolutionaryScale Team (ESM Team, 2024 ▸), available under the Cambrian Non-Commercial License at https://huggingface.co/EvolutionaryScale/esmc-600m-2024-12. Additional resources include the *DANCE* method (version of October 8 2024) developed by Lombard *et al.* (2024 ▸), available under the MIT license at https://github.com/PhyloSofS-Team/DANCE. As baselines, we ran the *NOLB* method (version 1.9) developed by Hoffmann & Grudinin (2017 ▸) and available at https://team.inria.fr/nano-d/software/nolb-normal-modes/, the *AlphaFlow* (version AlphaFlow-PDB distilled) and *ESMFlow* (version ESMFlow-PDB distilled) models developed by Jing, Berger *et al.* (2024 ▸) and available at https://github.com/bjing2016/alphaflow, and *BioEmu* developed by Lewis *et al.* (2025 ▸) available under the MIT license at https://github.com/microsoft/bioemu. We used *TM-align* (version 20220412) developed by Zhang & Skolnick (2005 ▸) and available at https://zhanggroup.org/TM-align/ to perform all-to-all pairwise structural alignments between train and test protein conformations and compute TM-scores.
